# Whole-Cell Fungal Biotransformation of *para*-Hydroxycinnamic Acids Mediated by Phenolic Acid Decarboxylase, Carboxylic Acid Reductase and Alcohol Dehydrogenase

**DOI:** 10.3390/molecules31152609

**Published:** 2026-07-27

**Authors:** Abirami Baskaran, Stefano Serra, El-Sayed R. El-Sayed, Tomasz Tronina, Jacek Łyczko, Teresa Olejniczak, Elisabetta Brenna, Filip Boratyński

**Affiliations:** 1 Department of Food Chemistry and Biocatalysis, Wrocław University of Environmental and Life Sciences, Norwida 25, 50-375 Wrocław, Poland; sayed_zahran2000@yahoo.com (E.-S.R.E.-S.); tomasz.tronina@upwr.edu.pl (T.T.); jacek.lyczko@upwr.edu.pl (J.Ł.); teresa.olejniczak@upwr.edu.pl (T.O.); 2 Istituto di Scienze e Tecnologie Chimiche “Giulio Natta”, Consiglio Nazionale delle Ricerche (SCITEC-CNR), Via Bassini 6, 20133 Milano, Italy; stefano.serra@cnr.it; 3 Plant Research Department, Nuclear Research Center, Egyptian Atomic Energy Authority, Cairo 11787, Egypt; 4 Dipartimento di Chimica, Materiali ed Ingegneria Chimica “Giulio Natta”, Politecnico di Milano, Piazza Leonardo da Vinci 32, 20133 Milano, Italy; mariaelisabetta.brenna@polimi.it

**Keywords:** endophytic fungi, biocatalysis, phenolic acid decarboxylase, alcohol dehydrogenase, *para*-hydroxycinnamic acid, 4-vinylphenol, 4-vinylguaiacol

## Abstract

Microbial biotransformation of *para*-hydroxycinnamic acids (*p*HCAs) such as *para*-coumaric, caffeic, ferulic and sinapic acids into vinylphenols is catalyzed by phenolic acid decarboxylases (PADs), while reduction to their corresponding aldehydes and alcohols is mediated by carboxylic acid reductases (CARs) and alcohol dehydrogenases (ADHs), respectively. The present study systematically evaluated a diverse set of endophytic and basidiomycetes fungi as whole-cell biocatalysts for the transformation of *p*HCAs into their corresponding vinylphenols and/or aldehydes and alcohols. Twenty-three fungal strains were screened for their PAD, CAR and ADH activities. Based on ultra-high-performance liquid chromatography–diode array detector (UHPLC-DAD) analysis, fourteen strains were selected for preparative-scale biotransformations across all four substrates. Previous literature largely emphasizes enzyme activity or single substrates, seldom covering all four *p*HCAs. The strain *Umbelopsis* sp. JAR-T demonstrated promising biotransformation of *para*-coumaric acid and ferulic acid to 4-vinylphenol (28% isolated yield) and 4-vinylguaiacol (40% isolated yield), respectively, with minimal by-product formation. The results highlight endophytic fungi as largely untapped and versatile biocatalysts for *p*HCA biotransformation and establish whole-cell fungal systems as robust, non-recombinant alternatives to engineered platforms. This integrated screening-to-preparative workflow provides a scalable framework for the production of value-added compounds with potential applications in the food, cosmetic and pharmaceutical industries.

## 1. Introduction

*para*-Hydroxycinnamic acids (*p*HCAs) are a class of naturally abundant, simple phenolic acids possessing a single aromatic ring as their core with a C6-C3 phenylpropanoid structure [1]. Produced as specialized metabolites (traditionally termed secondary metabolites) in plants and fungi, *p*HCAs protect against UV, defend against pathogens and influence flavor, smell and color of plants [2]. The most abundant natural *p*HCAs are *para*-coumaric acid (4-hydroxycinnamic acid, *p*CA), caffeic acid (3,4-dihydroxycinnamic acid, CA), ferulic acid (4-hydroxy-3-methoxycinnamic acid, FA) and sinapic acid (3,5-dimethoxy-4-hydroxycinnamic acid, SA) [3]. These compounds are synthesized inside the plastids and cytosol of plants through the shikimate and phenylpropanoid pathways, where phenylalanine serves as the precursor for the synthesis of *p*CA, CA, FA and SA along with lignin [4,5]. Although these acids are present in nature, their alcohols, aldehydes and vinyl derivatives remain scarce and relatively understudied. This scarcity has directed interest in sustainable production strategies, especially biological routes capable of selective structural modification.

**Figure 1:**
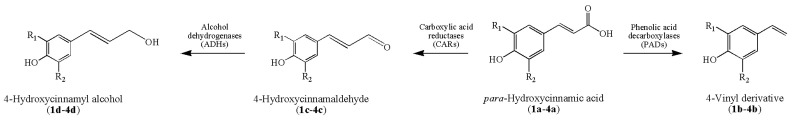
Products of PAD-, CAR- and ADH-mediated transformations of *p*HCA substrates: R_1_ = R_2_ = H (*p*-coumaric, **1**), R_1_ = OH and R_2_ = H (caffeic, **2**), R_1_ = OCH_3_ and R_2_ = H (ferulic, **3**), R_1_ = R_2_ = OCH_3_ (sinapic, **4**).

Microbial transformation employs whole cells of microorganisms or isolated enzymes to structurally modify phytochemicals and other organic compounds via biochemical reactions, facilitating the efficient production of valuable compounds. Compared to conventional chemical synthesis, biotransformation avoids harsh reaction environments and minimizes unwanted effects [6]. This environmentally friendly approach supports green chemistry for converting *p*HCAs into value-added derivatives for pharmaceutical and industrial use. Typically, enzymes present in microorganisms that aid in the *p*HCAs biotransformation include Phenolic Acid Decarboxylase (PAD), which mediates the decarboxylation of *p*HCAs to form their respective vinyl derivatives, and Carboxylic Acid Reductase (CAR) which catalyzes the ATP- and NAD(P)H-dependent reduction of *p*HCAs into their corresponding aldehydes and thereby to their alcohols, by means of Alcohol Dehydrogenase (ADH) catalysis. This microbial route bypasses the CoA activation and cinnamoyl-CoA reductase (CCR)-mediated reduction steps present in the canonical plant pathway, as fungal CARs directly reduce free hydroxycinnamic acids to aldehydes. These compounds are considered high-value products owing to their limited commercial availability and applications across broad range industries [7]. Figure 1 illustrates the biotransformation pathways of *p*HCAs.

Vinyl derivatives of *p*HCAs have diverse applications in addition to their food and pharmaceutical applications. 4-vinylphenol is used in semiconductors, photoresists and photolithography [8,9]; 4-vinylcatechol is utilized in the synthesis of polystyrenes, plastics and styrene-butadiene rubbers [9,10]; 4-vinylguaiacol is employed as a precursor in biocatalytic reactions to produce valuable food flavor compounds, such as ethylguaiacol, acetovanillone and vanillin, and its market value is approximately 30–40 times higher than FA, making it economically attractive [7]; and 4-vinylsyringol exhibits antioxidant and antimutagenic properties with potential applications in food processing, cosmetics and pharmaceuticals [11]. Beyond vinyl derivatives, hydroxycinnamyl alcohols are important intermediates in lignin biosynthesis, contributing to plant structure, water transport and pathogen defense; and they serve as valuable precursors for the sustainable production of polymers, resins, specialty chemicals and fragrance compounds, including those used in jasmine, gardenia and rose aromas. Their corresponding hydroxycinnamaldehydes are key intermediates in the synthesis of fine chemicals, flavors, fragrances and bioactive molecules, while also functioning as key metabolites in engineered microbial pathways [12,13]. Despite their high commercial value and broad applicability, the availability of these compounds remains limited because they are typically present in low concentrations in natural sources, while chemical synthesis often requires harsh reaction conditions and lacks the selectivity needed to produce natural-label products. Consequently, biotransformation of *p*HCAs into these derivatives offers a sustainable route to high-value chemicals for food, pharmaceutical, cosmetic, polymer and fine chemical applications, while increasing the economic value of renewable biomass-derived feedstocks.

Previous studies have reported biotransformation of one or more *p*HCAs through recombinant or enzyme-based strategies [9,14,15] as well as exploiting wild-type-based bacterial biotransformations [16]. Such investigations utilize bacteria engineered to express PAD, TAL (Tyrosine ammonia-lyase) and related decarboxylases to selectively produce high-titer single products. Few studies also involved yeasts and fungi, still focusing on enzyme activity in recombinant hosts with single-substrate transformation [17,18]. While effective for pathway optimization, these systems often require extensive genetic modification and external precursors, increasing process complexity. In contrast, whole-cell biocatalysts provide a self-sustaining catalytic environment in which enzymes remain in their native intracellular context, enabling cofactor regeneration and reducing the need for enzyme purification or supplementation [6]. Although isolated enzymes generally offer higher reaction specificity and simpler process control, whole-cell systems are particularly advantageous for multistep biotransformations involving sequential or competing enzymatic activities, making them attractive platforms for the conversion of *p*HCAs into structurally diverse derivatives. Fungi are known to inherently possess complex and compartmentalized metabolic networks, effective in catalyzing reduction, decarboxylation and oxidation reactions within a single cell. The handful of reports investigating ascomycetes [6,18,19] and basidiomycetes [20,21,22] have also focused on enzyme activity or single-substrate transformation, rather than exploring all four *p*HCA substrates or pathways leading to vinyl derivatives, aldehydes and alcohols. Compared with commonly used commercial fungal biocatalysts, endophytic fungi represent a promising resource. These fungi reside asymptomatically within plant tissues [23], where they are exposed to phenylpropanoid metabolites, suggesting a natural adaptation for metabolizing *p*HCAs and related aromatic compounds. In spite of this ecological relevance, they are largely unexplored for whole-cell biotransformation of *p*HCAs. One study explores the potential of endophyte *Phomopsis liquidambari* in the remediation of sinapic acid by converting it into 4-vinylsyringol [24]. While *p*CA, CA and FA are relatively studied, the gap in research is particularly evident with SA owing to its structural complexity. Beyond the study involving *P. liquidambari*, recent work has demonstrated sinapic acid conversion to 4-vinylsyringol using recombinant systems based on *Neolentinus lepideus* [22]. These isolated studies highlight the lack of systematic investigation into sinapic acid metabolism, particularly within native, whole-cell fungal systems.

The present study aimed to investigate a diverse set of endophytic and basidiomycete fungi as whole-cell biocatalysts for the transformation of *p*CA, CA, FA and SA. This study provides a comprehensive assessment of fungal PAD, CAR and ADH activities across multiple substrates by integrating screening-scale studies with preparative-scale biotransformation, ultra-high-performance liquid chromatography–diode array detector (UHPLC-DAD) analysis, flash purification and NMR-based structural confirmation. This study establishes a robust framework to develop sustainable, non-recombinant bioprocesses for the valorization of *p*HCAs.

## 2. Results

Biotransformation of *para*-hydroxycinnamic acids (*p*HCAs) using whole cells of microorganisms is a significant area of research beneficial in bioremediation, valorization of lignin and sustainable production of value-added phenolic compounds. In particular, fungi possess numerous enzymatic systems that aid in decarboxylation, reduction and other transformation reactions. Within this cluster, phenolic acid decarboxylase (PAD), carboxylic acid reductase (CAR) and alcohol dehydrogenase (ADH) activities gain particular emphasis, as they enable the conversion of *p*HCAs into vinylphenols, hydroxycinnamaldehydes and corresponding alcohols of industrial and biological relevance. The structures of the substrates and expected products through PAD, CAR and ADH pathways are depicted in Table 1.

### 2.1. Biotransformation at Screening Scale

A total of sixteen endophytic fungi and seven basidiomycetes were evaluated as whole-cell biocatalysts for the biotransformation of four *p*HCAs: *p*-coumaric acid (*p*CA), caffeic acid (CA), ferulic acid (FA) and sinapic acid (SA). These preliminary screening experiments were performed in Erlenmeyer flasks containing Sabouraud broth media, with each substrate added at a concentration of 50 mg per 100 mL of medium. Substrate conversion and product formation were monitored at regular time intervals initially using Gas Chromatography with Flame Ionization Detection (GC-FID) and Gas Chromatography–Mass Spectrometry (GC-MS). However, as the substrates underwent thermal degradation and did not yield reliable quantitative results, the analysis was performed using Ultra-High-Performance Liquid Chromatography–Diode Array Detector (UHPLC-DAD). As the same degradation pattern was detected for pure standards in the absence of fungal biomass, the phenomenon was attributed to thermal instability of the hydroxycinnamic acid substrates during GC analysis rather than to biological degradation. The fungal strains revealed distinctive, substrate-dependent biotransformation profiles. Based on dominant enzymatic routes, strains were classified according to PAD, CAR and ADH activity, or alternative oxidative and specialized metabolite pathways. Fourteen strains, comprising eleven endophytes and three basidiomycetes, exhibited consistent PAD and/or CAR and/or ADH activity towards at least one substrate and were selected for further scale-up to preparative-scale investigation (Table 2).

In the biotransformation of *p*-coumaric acid (**1a**), six strains (*Trichoderma harzianum* BUK-T, *Coniochaeta velutina* SW-B, *Umbelopsis* sp. JAR-T, *Diaporthe* sp. MOD-B, *Diaporthe eres* POP-L1 and *Penicillium chrysogenum* SOS-B2) formed 4-vinylphenol (**1b**), while five strains (*Diaporthe* sp. MOD-B, *D. eres* POP-L1, *Trametes hirsuta* d28, *Phanerochaete chrysosporium* KKP 784 and *Laetiporus sulphureus* AM 515) exhibited CAR and ADH activity. Two fungal strains, *Diaporthe* sp. MOD-B and *D. eres* POP-L1, uniquely displayed PAD, CAR and ADH activities, indicating metabolic branching between decarboxylation and reduction pathways. Biotransformation with caffeic acid (**2a**) exhibited higher selectivity with only three strains (*T. harzianum* BUK-T, *Aspergillus westerdijkiae* 17P and *Umbelopsis* sp. JAR-T) producing 4-vinylcatechol (**2b**) and five strains (*Diaporthe* sp. MOD-B, *D. eres* POP-L1, *T. hirsuta* d28, *P. chrysosporium* KKP 784 and *L. sulphureus* AM 515) showing CAR- and ADH-mediated product formation. In addition, *Chaetomium cochliodes* KLON-L1, based on GC-MS analysis, produced 4-vinyl-1-cyclohexene diepoxide. Biotransformation of ferulic acid (**3a**) yielded substantial metabolic diversity. Six strains (*T. harzianum* BUK-T, *A. westerdijkiae* 17P, *Umbelopsis* sp. JAR-T, *Diaporthe* sp. MOD-B, *D. eres* POP-L1 and *P. chrysogenum* SOS-B2) exhibited PAD activity, while seven strains (*T. harzianum* BUK-T, *A. westerdijkiae* 17P, *D. eres* POP-L1, *Umbelopsis* sp. 42, *T. hirsuta* d28, *L. sulphureus* AM 515 and *P. chrysosporium* KKP 784) demonstrated CAR and ADH activity. Several isolates, including *T. harzianum* BUK-T, *A. westerdijkiae* 17P and *D. eres* POP-L1, showed concurrent PAD, CAR and ADH activities, resulting in mixed product profiles. Additional oxidative metabolites, such as vanillic and isovanillic acids determined by GC-MS, were also detected in *Aspergillus ochraceus* ROB-L1 and *Penicillium yarmokense* JAR-L1 indicating the involvement of auxiliary metabolic pathways. Among all the *p*HCAs, biotransformation of sinapic acid (**4a**) was the most selective. Only two strains (*T. harzianum* BUK-T and *P. yarmokense* JAR-L1) showed PAD activity, while six strains (*T. harzianum* BUK-T, *Umbelopsis* sp. JAR-T, *Diaporthe* sp. MOD-B, *D. eres* POP-L1, *L. sulphureus* AM 515 and *P. chrysosporium* KKP 784) demonstrated CAR- and ADH-mediated reduction. *T. harzianum* BUK-T was the only strain to consistently exhibit PAD, CAR and ADH activity towards this substrate. For *T. harzianum* BUK-T and *P. yarmokense* JAR-L1, both strains were observed to produce additional metabolites such as syringic acid and acetosyringone beyond PAD, CAR and ADH pathways.

Overall, *T. harzianum* BUK-T exhibited the broadest substrate range and highest metabolic versatility in the screening study. *Diaporthe* sp. MOD-B and *D. eres* POP-L1 consistently demonstrated CAR and ADH activity and, in some cases, concurrent PAD activity. Basidiomycete strains, namely, *T. hirsuta* d28, *P. chrysosporium* KKP 784 and *L. sulphureus* AM 515, were predominantly associated with CAR- and ADH-mediated reduction. These findings provide a strong foundation for selecting fungal candidates for further preparative-scale biotransformation.

### 2.2. Biotransformation at Preparative Scale

Following preliminary screening, fourteen selected strains were evaluated under preparative-scale conditions to assess the scalability, efficiency and robustness of fungal whole-cell transformations of each *p*HCAs. Product formation and substrate depletion were quantified using UHPLC-DAD time-course analysis, and the conversion percentages for each substrate are summarized in Table 3, Table 4, Table 5 and Table 6.

Out of nine strains selected in the screening scale for biotransformation of *p*-coumaric acid (**1a**), three strains, namely, *Umbelopsis* sp. JAR-T, *Diaporthe* sp. MOD-B and *P. chrysogenum* SOS-B2, consistently produced **1b**. *Umbelopsis* sp. JAR-T achieved almost 88% substrate conversion within two days and maintained >98% **1b** abundance on the seventh day, indicating highly stable and dominant PAD activity. Although ADH-mediated reduction was observed in all three strains, it was subordinate to their PAD activity in the strains *Umbelopsis* sp. JAR-T and *P. chrysogenum* SOS-B2. Product formation of more than 50% of **1d** was demonstrated by *Diaporthe* sp. MOD-B, indicating that this strain can efficiently utilize both PAD and ADH pathways.

In the case of caffeic acid (**2a**), nine strains were selected in the screening study. On the preparative scale, only *C. cochliodes* KLON-L1 produced a measurable amount of **2b** (31%), and even with this strain, decarboxylation competed with the formation of **2d** and other metabolites, resulting in a mixture of reaction products. Formation of product **2d** was limited to transformations catalyzed by *C. cochliodes* KLON-L1 (11%) and *Diaporthe* sp. MOD-B (71%).

For ferulic acid (**3a**), PAD activity was more widely observed when compared with other substrates. With twelve strains shortlisted in screening, four strains, notably, *A. westerdijkiae* 17P (11%), *Umbelopsis* sp. JAR-T (100%), *Diaporthe* sp. MOD-B (22%) and *P. chrysogenum* SOS-B2 (32%), efficiently converted **3a** into **3b** in the preparative study. Among these, *Umbelopsis* sp. JAR-T demonstrated rapid, quantitative conversion with minimal side-product formation. Substrate **3a** also supported strong ADH and CAR activity. *A. westerdijkiae* 17P (75% of **3d** and 8% of **3c**) and *P. chrysogenum* SOS-B2 (46% of **3d**) produced substantial quantities of **3d**, often exceeding **3b** formation. This further supports **3a** as a highly compliant substrate for decarboxylative, reductive and dehydrogenative fungal biotransformations.

Regarding sinapic acid (**4a**), of the seven strains selected in the screening study, only *T. harzianum* BUK-T mediated the formation of **4d** (69%) as the dominant product of ADH activity.

The screening study identified a broad range of fungi capable of PAD-mediated decarboxylation, CAR-mediated reduction and ADH-mediated dehydrogenation of *p*HCAs. However, only a subset of these strains retained the stable and effective PAD, CAR and ADH pathways under preparative conditions. Under screening conditions, *T. harzianum* BUK-T and *D. eres* POP-L1 displayed high potential in transforming all four substrates under PAD and/or CAR/ADH pathways, yet, the same strains could not sustain scale-up, resulting in *T. harzianum* BUK-T demonstrating ADH activity only with **4a**. All three basidiomycete strains, *T. hirsuta* d28, *P. chrysosporium* KKP784 and *L. sulphureus* AM 515, despite exhibiting promising CAR and ADH activities under screening scale, unfavorably showed no measurable results. While *C. cochliodes* KLON-L1 was chosen at the screening scale for its metabolite production ability, it demonstrated a significant level of PAD activity with **2a** in the preparative scale. With **3a**, although *Umbelopsis* sp. JAR-T and *Umbelopsis* sp. 42 belong to the same genus, their catalytic abilities via the PAD, CAR and ADH systems differ markedly. *Umbelopsis* sp. JAR-T exhibited PAD activity, converting 100% of **3a** to **3b** at the preparative scale, whereas *Umbelopsis* sp. 42 showed CAR and ADH activities only at the screening scale. In addition, *Umbelopsis* sp. JAR-T also demonstrated prominent PAD activity with **1a** resulting in the formation of almost 100% of **1b**. The comparison highlights the necessity of moving beyond analytical screening to identify fungal biocatalysts capable of efficient, scalable conversion of *p*HCAs. Compound **3a** emerged as the most reliable substrate across both screening and preparative studies, while biotransformations with **2a** and **4a** remained challenging.

The reproducibility of the biotransformation was evaluated using *Umbelopsis* sp. JAR-T. The conversion of **1a** to **1b** and **3a** to **3b** reached 90.47 ± 4.81% and 93.54 ± 1.95%, respectively (mean ± SD, *n* = 3), after 7 and 2 days of incubation. Supplementary Figures S5 and S6 show the corresponding UHPLC-DAD chromatograms. The relatively low standard deviations observed for both transformations indicate good reproducibility among independent experiments and suggest consistent decarboxylation performance of the fungal whole-cell catalyst.

Quantitative assessment of recoverable products was achieved through purification of preparative-scale biotransformation extracts. With UHPLC-DAD guiding the product detection and flash chromatography determining final isolated yields, NMR analysis confirmed the product structures. In biotransformation of **1a**, *Umbelopsis* sp. JAR-T showed near-complete PAD-driven conversion to **1b** (Supplementary Figure S7) with an isolated yield of 28% after purification, identifying this strain as a highly efficient and selective PAD biocatalyst. In contrast, *Diaporthe* sp. MOD-B showed distributed metabolic flux between PAD and ADH pathways. Moreover, NMR analysis of the PAD-derived fraction also revealed the presence of ethylphenol. For biotransformation of **3a**, *Umbelopsis* sp. JAR-T yielded a highly selective recovery of **3b** (Supplementary Figure S8) with a 40% isolated yield, highlighting strong PAD activity and suitability for preparative-scale production. With *A. westerdijkiae* 17P and *P. chrysogenum* SOS-B2, coniferyl aldehyde-type structures were confirmed by NMR analysis, suggesting CAR as the predominant pathway.

## 3. Discussion

Most of the available literature studying *p*HCA biotransformation into its corresponding vinyl derivatives, alcohols and aldehydes has relied on isolated enzymes, recombinant microorganisms or wild-type bacterial strains. In particular, expression systems involving tyrosine ammonia-lyase (TAL), phenolic acid decarboxylase (PAD), carboxylic acid reductase (CAR) and related enzymes have been reported in bacterial host species like *Escherichia coli* [10,25,26], *Bacillus* [27,28,29,30,31] and *Streptomyces* [32,33]. Despite being effective in attaining high yield, these approaches predominantly require synthetic growth media and/or externally supplemented cofactors in addition to optimized expression systems and genetic modifications. Only a few studies [16,27,34] have directly employed bacteria in the biotransformation of one or more *p*HCAs into their respective vinyl derivatives. In line with these studies, the present work utilizes wild-type (non-GMO) fungal strains as biocatalysts.

Regardless of the comprehensive literature dealing with the PAD, CAR and ADH activities of bacteria, only a few studies have explored yeast [17,34,35,36] and fungal enzymes [19,21,22] as biocatalysts for the targeted biotransformation of *p*HCAs. Prevailing reports on fungal biotransformation consistently focus on recombinant enzymatic activity, typically employing purified enzymes instead of considering fungi as scalable, whole-cell production platforms. Furthermore, the existing studies have targeted ligninolytic and white-rot basidiomycetes, such as *Neolentinus lepideus* [22], *Trametes versicolor* [20], *Schizophyllum commune* [21], *Phanerochaete chrysosporium* [37] and *Trametes hirsuta* [37], as well as ascomycetes such as *Isaria farinosa* [18] and *Aspergillus luchuensis* [19] with investigations generally emphasizing individual substrates, specific enzymatic activities, or degradation pathways rather than systematic comparison of all four *p*HCAs and their corresponding vinyl, aldehyde and alcohol derivatives. Two research works show that the endophytic fungus *Phomopsis liquidambari* can metabolize both sinapic [24] and ferulic [38] acids through distinct pathways.

While **1a** and **3a** have been extensively investigated in microbial systems [17,18,19,25,34,39], with few systems exploring **2a** [10,20,34,35,40], the research gap is remarkably noticeable in biotransformations involving **4a**. Among the documented research, one study reports the conversion of **4a** to yield low concentrations of **4b** by employing the endophytic fungus, *Phomopsis liquidambari* [24]. Another study conducted recently described the biotransformation of **4a** to **4b** using recombinant *Neolentinus lepideus* [22]. These scarce available reports emphasize the lack of systematic investigation of **4a** metabolism, specifically employing whole-cell fungi.

Addressing the gap in prior research, the present study establishes a strategy to utilize whole fungal cells as biocatalysts for the conversion of *p*HCAs into their corresponding vinyl derivatives, aldehydes and alcohols without genetic engineering. The observed product profiles are consistent with sequential decarboxylation (PAD) and reduction (CAR and ADH) reactions, although the specific enzymes responsible were not investigated in the present study. Whole-cell systems promote the constant intracellular regeneration of cofactors required for enzyme activity. Moreover, cellular compartmentalization in fungi enables the simultaneous functioning of multiple enzymatic pathways, thereby facilitating parallel decarboxylation and reduction processes within a single organism. Recombinant bacterial platforms often lack this metabolic integration because they are generally optimized for the production of a single dominant product [39,41]. The present work extends this narrow knowledge base by meticulously screening and scaling a diverse set of fungal strains belonging to endophytes and basidiomycetes, illustrating that several strains can achieve product selectivity and yields relative to those reported for the engineered bacterial systems.

The focus on endophytic fungi as whole-cell biocatalysts is another novel aspect of this study. Apart from the solitary report by Xie et al. [24], few studies have investigated endophytes for the production of vinyl derivatives or hydroxycinnamic alcohols. Since endophytes inhabit plant tissues, natural exposure to phenylpropanoid-derived metabolites is inevitable suggesting an intrinsic evolutionary adaptation for processing hydroxycinnamic acids and related compounds. This ecological hypothesis is supported by the strong PAD, CAR and ADH activities observed in the present study across diverse endophytic fungal species, establishing them as a largely untapped resource as biocatalysts.

Correspondingly, the results from UHPLC-DAD analysis and flash purification facilitated the quantification of biotransformation within whole-cell fungal systems. To verify the consistency of biotransformation performance, the selected high-yield strain *Umbelopsis* sp. JAR-T was grown in triplicate for the biotransformation of **1a** and **3a**. Comparable substrate conversion rates and product yields were observed across all replicates, indicating stable biotransformation capability. The attained yields did not differ substantially from those reported in recombinant bacterial systems [27,30,31], at the same time avoiding the operational and regulatory complexity associated with genetically modified organisms. Additionally, the wider metabolic profiles of endophytic strains *A. westerdijkiae* 17P and *Diaporthe* sp. MOD-B indicate the fungal capability in generating diverse products rather than targeting single-product reactions. Additionally, endophyte *T. harzianum* BUK-T was capable of converting **4a** into **4d** through the ADH pathway, albeit with limited efficiency.

## 4. Materials and Methods

The substrates for the biotransformations—*para*-coumaric acid (≥98%) and caffeic acid (≥98.5%)—were purchased from Glentham Life Sciences (Corsham, UK), ferulic acid (≥99%) was purchased from Sigma Aldrich (St. Louis, MO, USA) and sinapic acid (98.8%) was purchased from Ambeed (Arlington Heights, IL, USA). The pattern compounds of the expected products of PAD activity, namely, 4-vinylphenol (10wt% in propylene glycol), was purchased from Merck (Darmstadt, Germany) and 4-vinylsyringol (98.5%) was purchased from MedChemExpress (Monmouth Junction, NJ, USA). The compounds 4-vinylcatechol and 4-vinylguaiacol were obtained via chemical synthesis [42] and purified using bulb-to-bulb distillation. The pattern compounds of the expected products of CAR and ADH activity, namely, *para*-coumaryl alcohol (98.4%), caffeoyl alcohol (99.9%), sinapyl alcohol (98.7%) and sinapyl aldehyde (99.9%), were purchased from MedChemExpress. Coniferyl alcohol (98%) and coniferyl aldehyde (98%) were purchased from Merck. Analytical-grade solvents such as methanol, ethyl acetate and DMSO were obtained from Sigma Aldrich, while salts such as anhydrous sodium sulfate was purchased from Chempur (Piekary Śląskie, Poland).

### 4.1. Microorganisms and Culture Maintenance

A total of twenty-three fungal strains, comprising sixteen endophytes and seven basidiomycetes, were used in this study. All fungal strains were maintained on Sabouraud Dextrose (peptone: 10 g/L, glucose: 30 g/L and agar: 20 g/L) agar (SDA) plates and stored at 4 °C.

Endophytic strains used in the biotransformation include the following: *Aspergillus ochraceus* ROB-L1, *Aspergillus westerdijkiae* 17P, *Aureobasidium pullulans* POP-L2, *Chaetomium cochliodes* KLON-L1, *Coniochaeta velutina* SW-B, *Diaporthe eres* POP-L1, *Diaporthe* sp. MOD-B, *Epicoccum nigrum* COR-B, *Monascus ruber* M22, *Penicillium chrysogenum* SOS-B2, *Penicillium yarmokense* JAR-L1, *Sphaeropsis sapinea* BUK-L2, *Trichoderma harzianum* BUK-T, *Umbelopsis isabellina* COR-L1, *Umbelopsis* sp. 42 and *Umbelopsis* sp. JAR-T. These strains were previously isolated from Mokrzański forest plants in Wrocław, Poland [43] and procured from the fungal repository maintained by the Department of Food Chemistry and Biocatalysis, Wrocław University of Environmental and Life Sciences, under the voucher BioExplor22.

Basidiomycete strains including *Inonotus radiatus* AM 70, *Laetiporus sulphureus* AM 515, *Phanerochaete chrysosporium* KKP 784, *Pholiota aurivella* AM 522 and *Trametes hirsuta* d28 were sourced from the fungal culture collection of the Department of Food Chemistry and Biocatalysis at the Wrocław University of Environmental and Life Sciences. *Agrocybe aegerita* DSM 22459 and *Pycnoporus cinnabarinus* DSM 15225 were obtained from Istituto di Scienze e Tecnologie Chimiche -Consiglio Nazionale delle Ricerche (SCITEC-CNR) (Milano, Italy).

### 4.2. Growth Medium and Inoculum Preparation

Sabouraud Dextrose broth was used as the basal medium for both screening and preparative-scale biotransformation experiments. Pre-cultures were generated by transferring two actively growing mycelial plugs (5 mm) into 100 mL of broth in 250 mL Erlenmeyer flasks, followed by incubation at 28 °C with orbital shaking at 120 rpm for 5–7 days.

### 4.3. Screening-Scale Biotransformation

Screening-scale biotransformations were carried out in 250 mL Erlenmeyer flasks containing 100 mL sterile Sabouraud Dextrose broth. Flasks were inoculated with 2 plugs of freshly grown fungi from agar plates [44] and incubated for 5–7 days at 28 °C in a shaking incubator. After incubation, 50 mg of substrates (either *p*CA, CA, FA or SA) dissolved in 1 mL of DMSO [6] was added aseptically to the culture medium and the samples were extracted at different time points between 1 and 7 days using ethyl acetate to determine the substrate depletion and product formation by TLC (thin layer chromatography), GC-FID (gas chromatography—flame ionization detection), GC-MS (gas chromatography—mass spectrometry) and UHPLC-DAD.

### 4.4. Preparative-Scale Biotransformation

Based on the screening study, fourteen fungi exhibiting consistent PAD and/or CAR and/or ADH activity were selected for preparative-scale biotransformation which was carried out in 2000 mL Erlenmeyer flasks containing 400 mL Sabouraud Dextrose broth with 25% (*v*/*v*) pre-culture. After incubation for 5–7 days at 28 °C in a rotary shaker, 200 mg of each *p*HCA dissolved in 4 mL of DMSO was added to each fungal culture. Biotransformation progress was monitored by collecting samples at different time points within 1–7 days, extracted using ethyl acetate, dried over anhydrous Na_2_SO_4_, evaporated using a vacuum rotary evaporator (Heidolph, Schwabach, Germany), diluted and dissolved in methanol for UHPLC-DAD analysis.

Based on the progress, the entire reaction mixture was extracted thrice using ethyl acetate, and the organic phase was dried over anhydrous Na_2_SO_4_ and concentrated using a vacuum rotary evaporator to obtain crude products. The products were then purified using flash chromatography and the structures of the individual compounds were determined using NMR (Nuclear Magnetic Resonance) analysis.

The screening- and preparative-scale experiments were performed once for all fungal strains. To assess reproducibility, the highest-performing strain was subsequently evaluated in three independent biological replicates. Conversion values are reported as mean ± SD (*n* = 3).

### 4.5. Analytical Methods

#### 4.5.1. Thin-Layer Chromatography (TLC)

The daily collected extracts were subjected to TLC fractionation (60 F254, Merck, Darmstadt, Germany) using hexane/ethyl acetate (1:1, *v*/*v*) as the eluent. The developed TLC plates were air-dried and examined using iodine vapor.

#### 4.5.2. Gas Chromatography–Flame Ionization Detection (GC-FID)

Analyses were conducted using an Agilent 7890A GC-FID (Agilent Technologies, Santa Clara, CA, USA) platform equipped with a non-polar DB-5MS column (30 m × 0.32 mm × 0.25 µm). The analytical method employed an injection port temperature of 250 °C and a programmed oven temperature: an initial 60 °C isotherm, a ramp to 325 °C at 10 °C min^−1^, and a 1 min terminal hold. Constant-flow H_2_ was utilized as the carrier gas. Relative distributions of products and remaining substrates were determined through the integration and subsequent normalization of the resulting chromatograms.

#### 4.5.3. Gas Chromatography–Mass Spectrometry (GC-MS)

Metabolite identification was performed using a GC-MS system, a Shimadzu QP 2020 Instrument (Shimadzu, Kyoto, Japan) equipped with column SH-Rxi-5SilMS (30 m × 0.25 mm × 0.25 µm). An amount of 1 μL of the sample was injected with splitless or split 10 modes, depending on sample concentration, at 260 °C. Helium was used as the carrier gas with a linear velocity of 36 cm·s^−1^. The GC initial temperature was 60 °C, held for 1 min, then ramped up to 325 °C at a rate of 10 °C ·min^−1^. The MS was operated in scan mode 40–600 *m*/*z*, with ion source and interface temperature 250 °C.

Compounds were tentatively identified by comparing mass spectra and retention indices with entries in the NIST mass spectral library and confirmed using authentic standards when available.

#### 4.5.4. Ultra-High-Performance Liquid Chromatography (UHPLC)

Ultra-high-performance liquid chromatography (UHPLC) was executed using an Ultimate 3000 UHPLC+ Focused system (Thermo Scientific, Waltham, MA, USA). Chromatographic separation was achieved on an Acclaim RSLC Polar Advantage II C18 column (2.1 mm × 100 mm, 2.2 µm, Thermo Scientific, Waltham, MA, USA), maintained at a constant temperature of 28 °C. The mobile phase consisted of 0.1% formic acid (HCOOH) in water (Solvent A) and 0.1% HCOOH in acetonitrile (Solvent B). A flow rate of 0.7 mL min^−1^ was employed with the following gradient profile: 0 to 5.0 min, linear gradient from 98% to 2% A; 5.0 to 6.0 min, isocratic hold at 2% A; 6.0 to 6.2 min, return to 98% A; and 6.2 to 8.0 min, re-equilibration at 98% A. Detection was performed from λ = 210 to λ = 450 nm with a data acquisition frequency of 25 Hz.

Retention times of the compounds are *t*_R_ *para*-coumaric acid: 3.65 min, *t*_R_ caffeic acid: 3.26 min, *t*_R_ ferulic acid: 3.56 min, *t*_R_ sinapic acid: 3.47 min, *t*_R_ 4-vinylphenol: 4.37 min, *t*_R_ 4-vinylcatechol: 3.84 min, *t*_R_ 4-vinylguaiacol: 4.19 min, *t*_R_ 4-vinylsyringol: 4.02 min, *t*_R_ *p*-coumaryl alcohol: 3.25 min, *t*_R_ caffeoyl alcohol: 2.87 min, *t*_R_ coniferyl alcohol: 3.22 min, *t*_R_ coniferyl aldehyde: 3.63 min, *t*_R_ sinapyl alcohol: 3.16 min and *t*_R_ sinapyl aldehyde: 3.54 min. Representative UHPLC-DAD chromatograms are shown in Supplementary Figures S1–S4.

#### 4.5.5. Flash Chromatography

Crude extracts were fractionated via automated flash chromatography utilizing a puriFlash® SX520 Plus system (Interchim, Montluçon, France). Separation was achieved on a silica gel stationary phase employing a stepwise gradient of hexane/ethyl acetate (from 9:1 to 1:1, *v*/*v*) as the mobile phase. Fractions were monitored by TLC and UHPLC-DAD, pooled based on chemical similarity, and concentrated to dryness. The isolated yields were recorded.

#### 4.5.6. Nuclear Magnetic Resonance (NMR)

Structural confirmation of compounds was performed using ^1^H and ^13^C NMR spectroscopy (Bruker Advance DRX 600 (600 MHz) spectrometer (Billerica, MA, USA)). Spectra were recorded in CD_3_OD (Methanol-d_4_). Chemical shifts were reported in parts per million (ppm) relative to the residual solvent signal. The NMR spectra of all analyzed compounds are given as follows:

*p-*coumaric acid (**1a**) ^1^H NMR (600 MHz, CD_3_OD) δ 7.56 (d, *J* = 15.9 Hz, 1H, H-7), 7.44–7.38 (m, 2H, H-2, H-6), 6.80–6.74 (m, 2H, H-3, H-5), 6.24 (d, *J* = 15.9 Hz, 1H, H-8). ^13^C NMR (151 MHz, CD_3_OD) δ 171.1 (C-9), 161.1 (C-4), 146.6 (C-7), 131.1 (C-2, C-6), 127.3 (C-1), 116.8 (C-3, C-5), 115.6 (C-8).

caffeic acid (**2a**) ^1^H NMR (600 MHz, CD_3_OD) δ 7.49 (d, *J* = 15.9 Hz, 1H, H-7), 7.00 (d, *J* = 2.1 Hz, 1H, H-2), 6.90 (dd, *J* = 8.2, 2.0 Hz, 1H, H-6), 6.74 (d, *J* = 8.2 Hz, 1H, H-5), 6.18 (d, *J* = 15.9 Hz, 1H, H-8). ^13^C NMR (151 MHz, CD_3_OD) δ 171.1 (C-9), 149.5 (C-4), 147.0 (C-7), 146.8 (C-3), 127.8 (C-1), 122.8 (C-6), 116.5 (C-5), 115.6 (C-8), 115.1 (C-2).

ferulic acid (**3a**) ^1^H NMR (600 MHz, CD_3_OD) δ 7.56 (d, *J* = 15.9 Hz, 1H, H-7), 7.14 (d, *J* = 2.0 Hz, 1H, H-2), 7.02 (dd, *J* = 8.2, 1.9 Hz, 1H, H-6), 6.77 (d, *J* = 8.2 Hz, 1H, H-5), 6.27 (d, *J* = 15.9 Hz, 1H, H-8), 3.85 (s, 3H, OCH_3_). ^13^C NMR (151 MHz, CD_3_OD) δ 171.0 (C-9), 150.5 (C-4), 149.4 (C-3), 146.9 (C-7), 127.8 (C-1), 124.0 (C-6), 116.5 (C-5), 115.9 (C-8), 111.7 (C-2), 56.4 (OCH_3_).

sinapic acid (**4a**) ^1^H NMR (600 MHz, CD_3_OD) δ 7.55 (d, *J* = 15.9 Hz, 1H, H-7), 6.86 (s, 2H, H-2, H-6), 6.30 (d, *J* = 15.8 Hz, 1H, H-8), 3.84 (s, 6H, 2×OCH_3_). ^13^C NMR (151 MHz, CD_3_OD) δ 170.9 (C-9), 149.5 (C-3, C-5), 147.1 (C-7), 139.5 (C-4), 126.7 (C-1), 116.4 (C-8), 106.9 (C-2, C-6), 56.8 (2×OCH_3_).

4-vinylphenol (**1b**) ^1^H NMR (600 MHz, CD_3_OD) δ 7.24–7.18 (m, 2H, H-2, H-6), 6.71–6.67 (m, 2H, H-3, H-5), 6.58 (dd, *J* = 17.6, 10.9 Hz, 1H, H-7), 5.51 (dd, *J* = 17.6, 1.1 Hz, 1H, H-8*_trans_*), 4.98 (dd, *J* = 10.9, 1.1 Hz, 1H, H-8*_cis_*). ^13^C NMR (151 MHz, CD_3_OD) δ 158.5 (C-4), 137.9 (C-7), 128.6 (C-1), 128.4 (C-2, C-6), 116.2 (C-3, C-5), 110.8 (C-8).

4-vinylcatechol (**2b**) ^1^H NMR (600 MHz, CD_3_OD) δ 6.87 (d, *J* = 1.8 Hz, 1H, H-2), 6.72–6.66 (m, 2H, H-5, H-6), 6.52 (dd, *J* = 17.6, 10.9 Hz, 1H, H-7), 5.48 (dd, *J* = 17.6, 1.1 Hz, 1H, H-8*_trans_*), 4.97 (dd, *J* = 10.9, 1.0 Hz, 1H, H-8*_cis_*). ^13^C NMR (151 MHz, CD_3_OD) δ 146.6 (C-4), 146.4 (C-3), 138.0 (C-7), 131.2 (C-1), 119.6 (C-6), 116.2 (C-5), 113.6 (C-2), 110.8 (C-8).

4-vinylguaiacol (**3b**) ^1^H NMR (600 MHz, CD_3_OD) δ 6.97 (d, *J* = 1.9 Hz, 1H, H-2), 6.81 (dd, *J* = 8.2, 1.9 Hz, 1H, H-6), 6.70 (d, *J* = 8.1 Hz, 1H, H-5), 6.58 (dd, *J* = 17.6, 10.9 Hz, 1H, H-7), 5.54 (d, *J* = 18.6 Hz, 1H, H-8*_trans_*), 5.01 (d, *J* = 11.9 Hz, 1H, H-8*_cis_*), 3.82 (s, 3H, OCH_3_). ^13^C NMR (151 MHz, CD_3_OD) δ 149.0 (C-3), 147.7 (C-4), 138.1 (C-7), 131.3 (C-1), 120.8 (C-6), 116.1 (C-5), 111.1 (C-8), 110.2 (C-2), 56.3 (OCH_3_).

4-vinylsyringol (**4b**) ^1^H NMR (600 MHz, CD_3_OD) δ 6.67 (s, 2H, H-2, H-6), 6.58 (dd, *J* = 17.6, 10.9 Hz, 1H, H-7), 5.57 (dd, *J* = 17.6, 0.9 Hz, 1H, H-8*_trans_*), 5.04 (dd, *J* = 10.9, 0.8 Hz, 1H, H-8*_cis_*), 3.81 (s, 6H, 2×OCH_3_). ^13^C NMR (151 MHz, CD_3_OD) δ 149.3 (C-3, C-5), 138.3 (C-7), 136.8 (C-4), 130.3 (C-1), 111.5 (C-8), 104.7 (C-2, C-6), 56.7 (2×OCH_3_).

*p-*coumaryl alcohol (**1d**) ^1^H NMR (600 MHz, CD_3_OD) δ 7.20 (d, *J* = 8.6 Hz, 2H, H-2, H-6), 6.68 (d, *J* = 8.6 Hz, 2H, H-3, H-5), 6.46 (d, *J* = 15.8 Hz, 1H, H-7), 6.13 (dt, *J* = 15.8, 6.0 Hz, 1H, H-8), 4.14 (dd, *J* = 6.0, 1.4 Hz, 2H, H-9). ^13^C NMR (151 MHz, CD_3_OD) δ 158.3 (C-4), 131.9 (C-7), 130.0 (C-1), 128.7 (C-2, C-6), 126.7 (C-8), 116.3 (C-3, C-5), 63.9 (C-9).

caffeoyl alcohol (**2d**) ^1^H NMR (600 MHz, CD_3_OD) δ 6.83 (d, *J* = 1.9 Hz, 1H, H-2), 6.70–6.63 (m, 2H, H-5, H-6), 6.40 (d, *J* = 15.8 Hz, 1H, H-7), 6.08 (dt, *J* = 15.8, 6.0 Hz, 1H, H-8), 4.13 (dd, *J* = 6.0, 1.4 Hz, 2H, H-9). ^13^C NMR (151 MHz, CD_3_OD) δ 146.4 (C-3), 146.3 (C-4), 132.2 (C-7), 130.7 (C-1), 126.7 (C-8), 119.9 (C-6), 116.3 (C-5), 113.9 (C-2), 63.9 (C-9).

coniferyl alcohol (**3d**) ^1^H NMR (600 MHz, CD_3_OD) δ 6.94 (d, *J* = 1.9 Hz, 1H, H-2), 6.79 (dd, *J* = 8.1, 1.9 Hz, 1H, H-6), 6.68 (d, *J* = 8.1 Hz, 1H, H-5), 6.45 (d, *J* = 15.8 Hz, 1H, H-7), 6.14 (dt, *J* = 15.8, 5.9 Hz, 1H, H-8), 4.14 (dd, *J* = 5.9, 1.3 Hz, 2H, H-9), 3.80 (s, 3H, OCH_3_). ^13^C NMR (151 MHz, CD_3_OD) δ 149.0 (C-3), 147.5 (C-4), 132.0 (C-7), 130.6 (C-1), 127.0 (C-8), 120.9 (C-6), 116.2 (C-5), 110.5 (C-2), 63.9 (C-9), 56.3 (OCH_3_).

coniferyl aldehyde (**3c**) ^1^H NMR (600 MHz, CD_3_OD) δ 9.52 (d, *J* = 7.9 Hz, 1H, H-9), 7.51 (d, *J* = 15.7 Hz, 1H, H-7), 7.19 (d, *J* = 1.9 Hz, 1H, H-2), 7.11 (dd, *J* = 8.2, 2.0 Hz, 1H, H-6), 6.81 (d, *J* = 8.2 Hz, 1H, H-5), 6.59 (dd, *J* = 15.7, 7.9 Hz, 1H, H-8), 3.85 (s, 3H, OCH_3_). ^13^C NMR (151 MHz, CD_3_OD) δ 196.2 (C-9), 156.2 (C-7), 151.7 (C-4), 149.5 (C-3), 127.6 (C-1), 126.7 (C-8), 125.1 (C-6), 116.6 (C-5), 112.1 (C-2), 56.5 (OCH_3_).

sinapyl alcohol (**4d**) ^1^H NMR (600 MHz, CD_3_OD) δ 6.66–6.63 (m, 2H, H-2, H-6), 6.45 (d, *J* = 15.8 Hz, 1H, H-7), 6.17 (dt, *J* = 15.8, 5.9 Hz, 1H, H-8), 4.15 (dd, *J* = 5.9, 1.5 Hz, 2H, H-9), 3.80 (s, 6H, 2x OCH_3_). ^13^C NMR (151 MHz, CD_3_OD) δ 149.3 (C-3, C-5), 136.6 (C-4), 132.2 (C-7), 129.7 (C-1), 127.5 (C-8), 104.9 (C-2, C-6), 63.8 (C-9), 56.7 (2×OCH_3_).

sinapyl aldehyde (**4c**) ^1^H NMR (600 MHz, CD_3_OD) δ 9.54 (d, *J* = 7.8 Hz, 1H, H-9), 7.54 (d, *J* = 15.7 Hz, 1H, H-7), 6.95 (s, 2H, H-2, H-6), 6.69 (dd, *J* = 15.7, 7.8 Hz, 1H, H-8), 3.85 (s, 6H, 2x OCH_3_). ^13^C NMR (151 MHz, CD_3_OD) δ 196.1 (C-9), 156.4 (C-7), 149.6 (C-3, C-5), 140.7 (C-4), 127.1 (C-8), 126.5 (C-1), 107.6 (C-2, C-6), 56.9 (2×OCH_3_).

## 5. Conclusions

This study moves beyond enzyme-centric and recombinant strategies towards the functional exploitation of endophytic fungi and basidiomycetes as whole-cell biocatalysts in the biotransformation of *p*HCAs. One of the most extensive comparative investigations of fungal PAD, CAR and ADH activities across four *p*HCA substrates, including the sparsely researched sinapic acid, is presented, concurrently implementing endophytes as a prospective resource with relatively untapped potential for sustainable valorization of *p*HCAs. Although the conversion efficiency was low, endophytic *T. harzianum* BUK-T was capable of transforming sinapic acid into sinapyl alcohol via the ADH pathway. In addition, *Umbelopsis* sp. JAR-T demonstrated efficient biotransformation of *p*-coumaric acid and ferulic acid into 4-vinylphenol and 4-vinylguaiacol, respectively, via the PAD pathway, with isolated yields of 28% and 40%. Integration of preparative-scale recovery with UHPLC-DAD monitoring and flash chromatography purification, the investigation described in this work connects essential enzymatic reactions with translational bioprocess development, delivering a robust approach with a reduced environmental footprint for applications currently addressed by engineered microbial systems.

## Figures and Tables

**Table 1:** Expected products from biotransformations of *p*HCA through PAD, CAR and ADH pathways.

No.	Substrate	Structure	PAD Pathway		CAR Pathway		ADH Pathway	
			Product	Structure	Product	Structure	Product	Structure
1.	*para*-coumaric acid (*p*CA, **1a**)	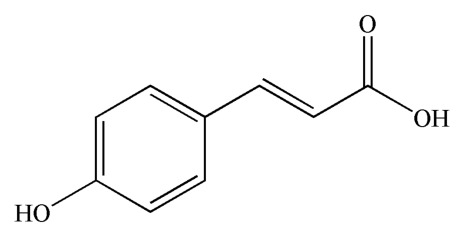	4-vinylphenol (4VP, **1b**)	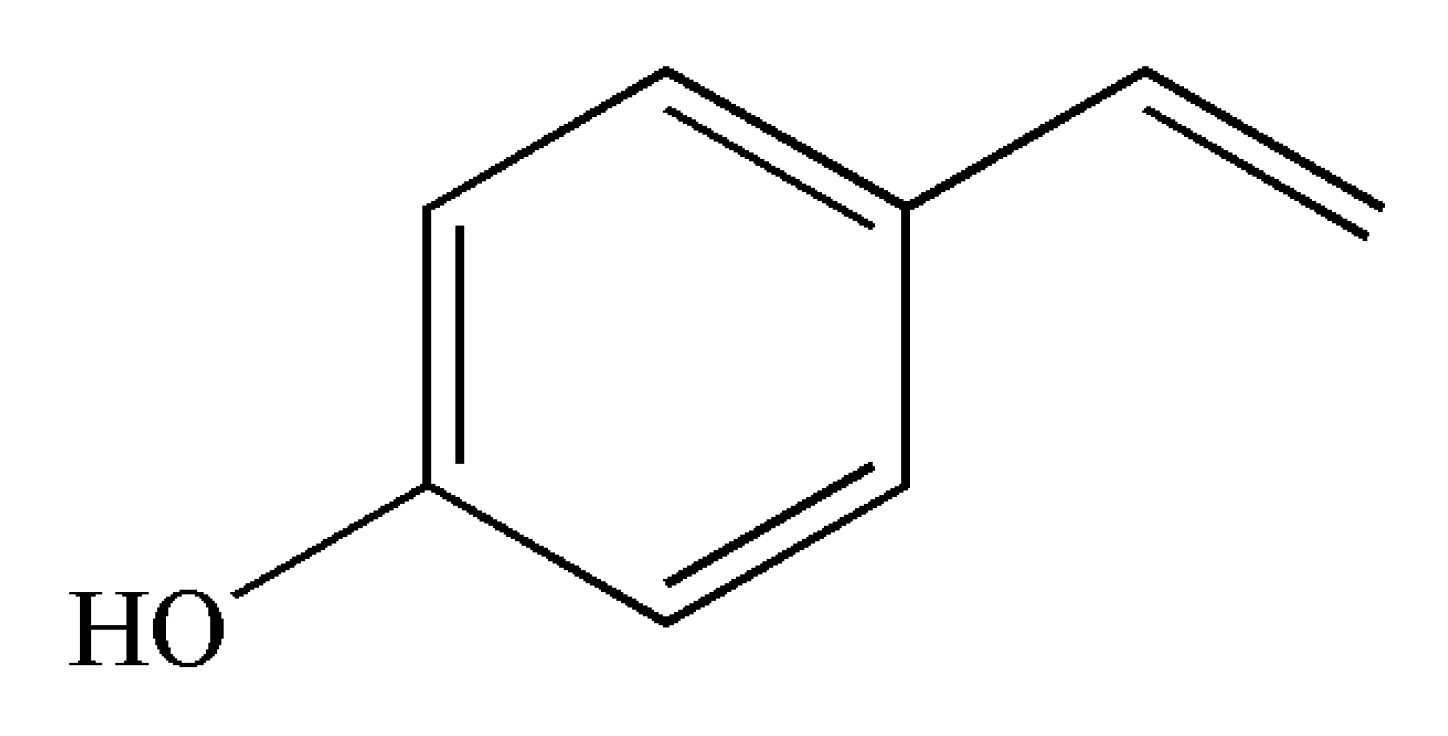	*para*-coumaryl aldehyde (**1c**)	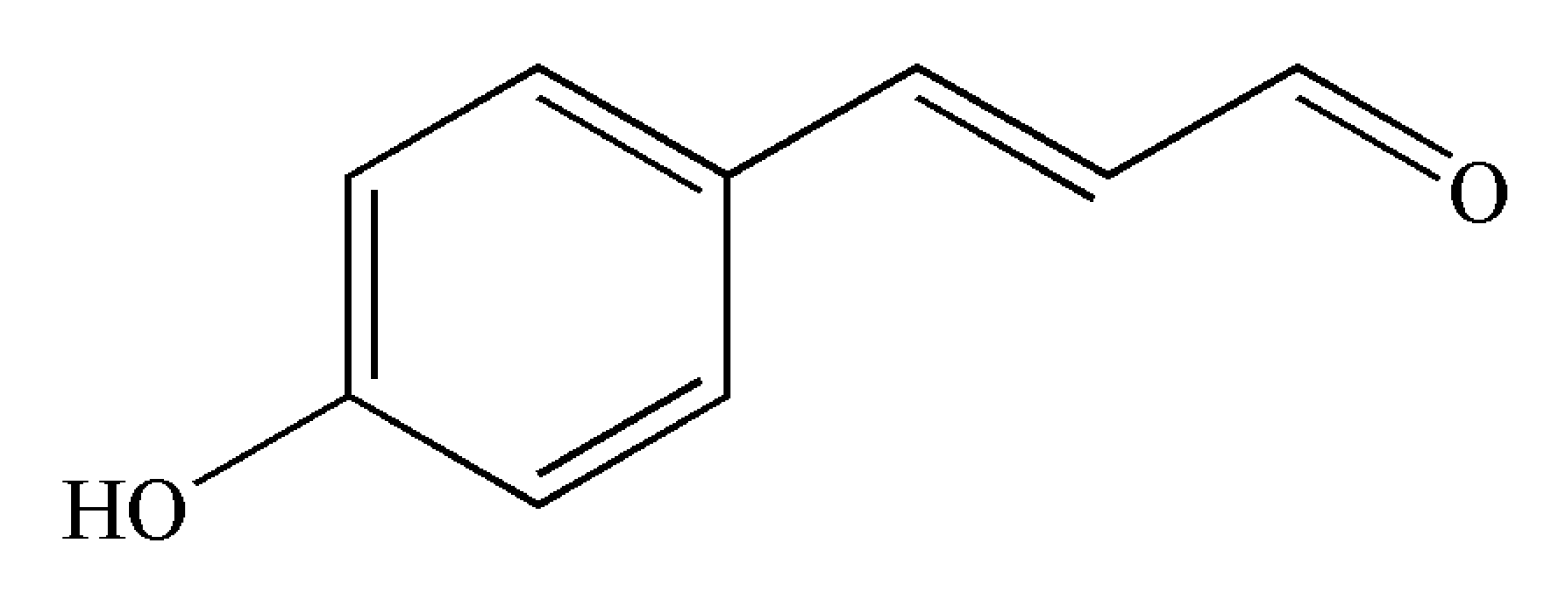	*para*-coumaryl alcohol (**1d**)	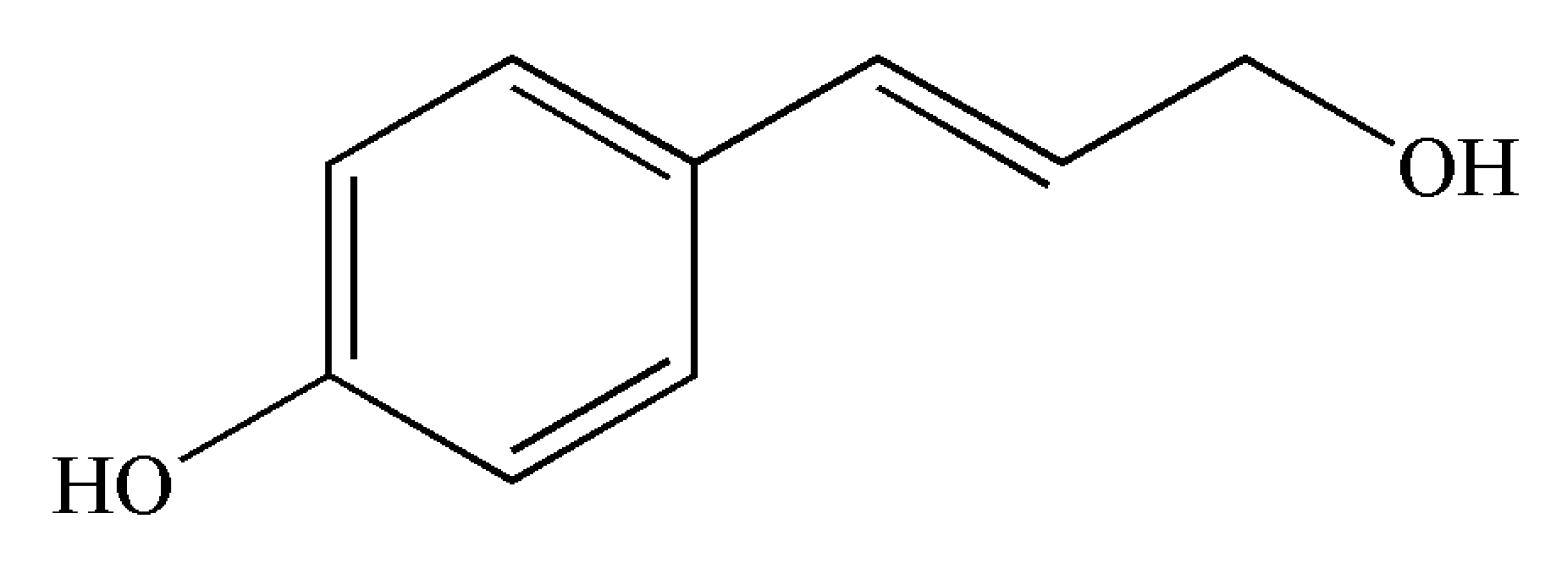
2.	Caffeic acid (CA, **2a**)	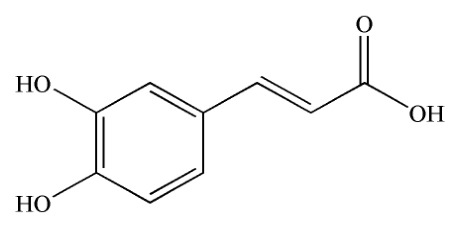	4-vinylcatechol (4VC, **2b**)	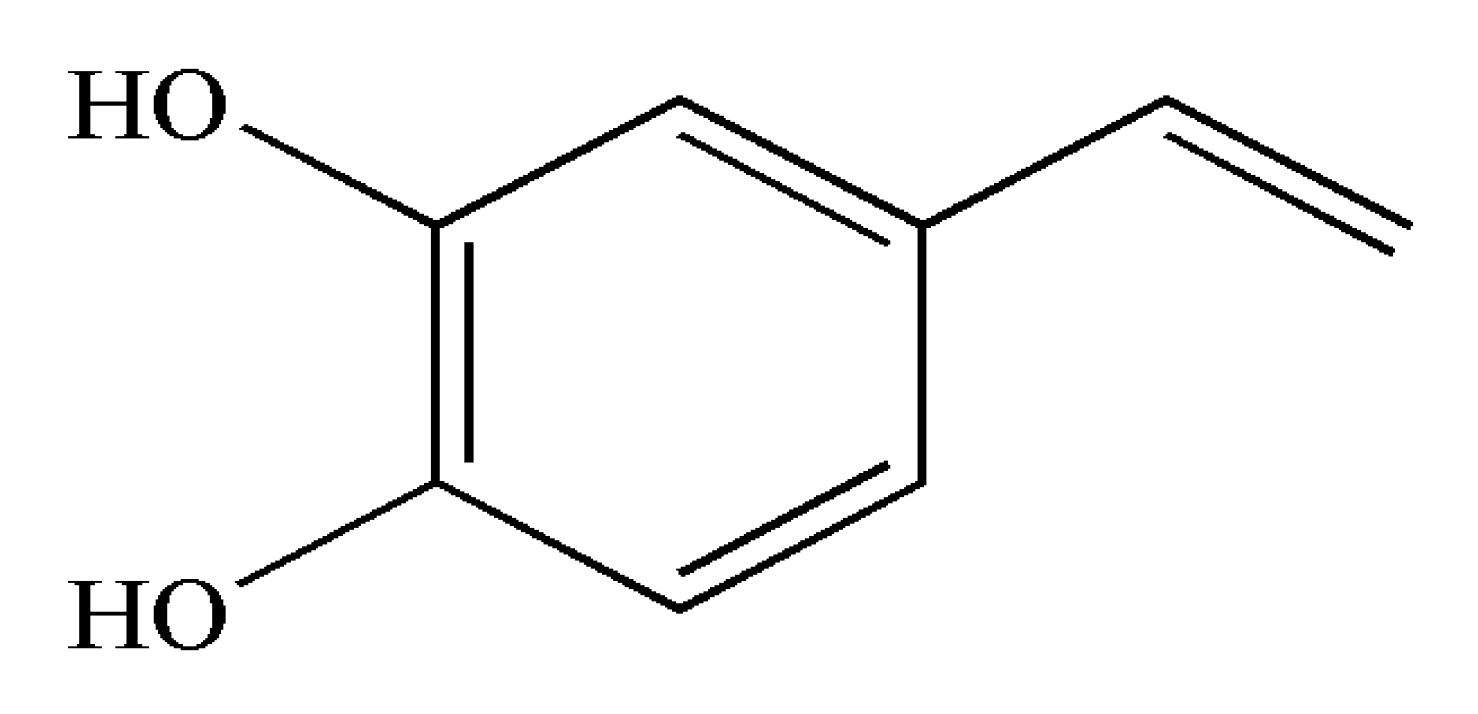	caffeoyl aldehyde (**2c**)	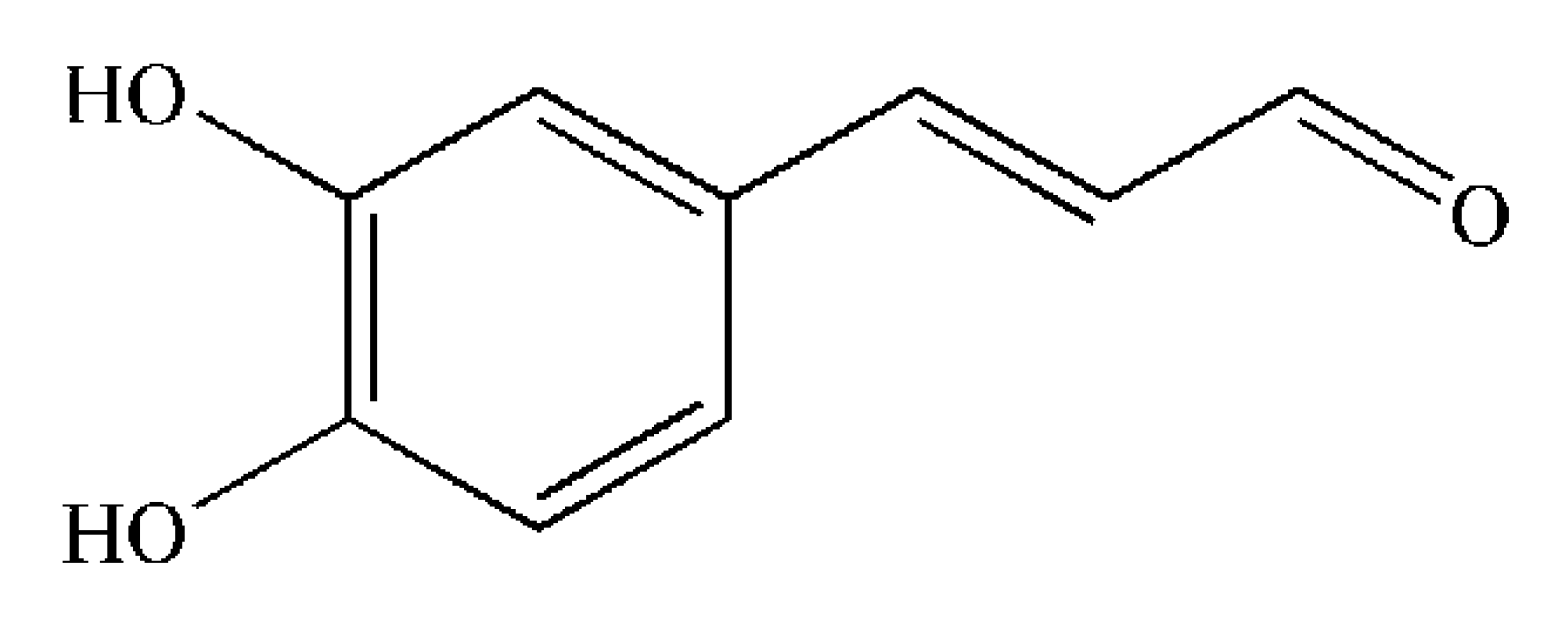	caffeoyl alcohol (**2d**)	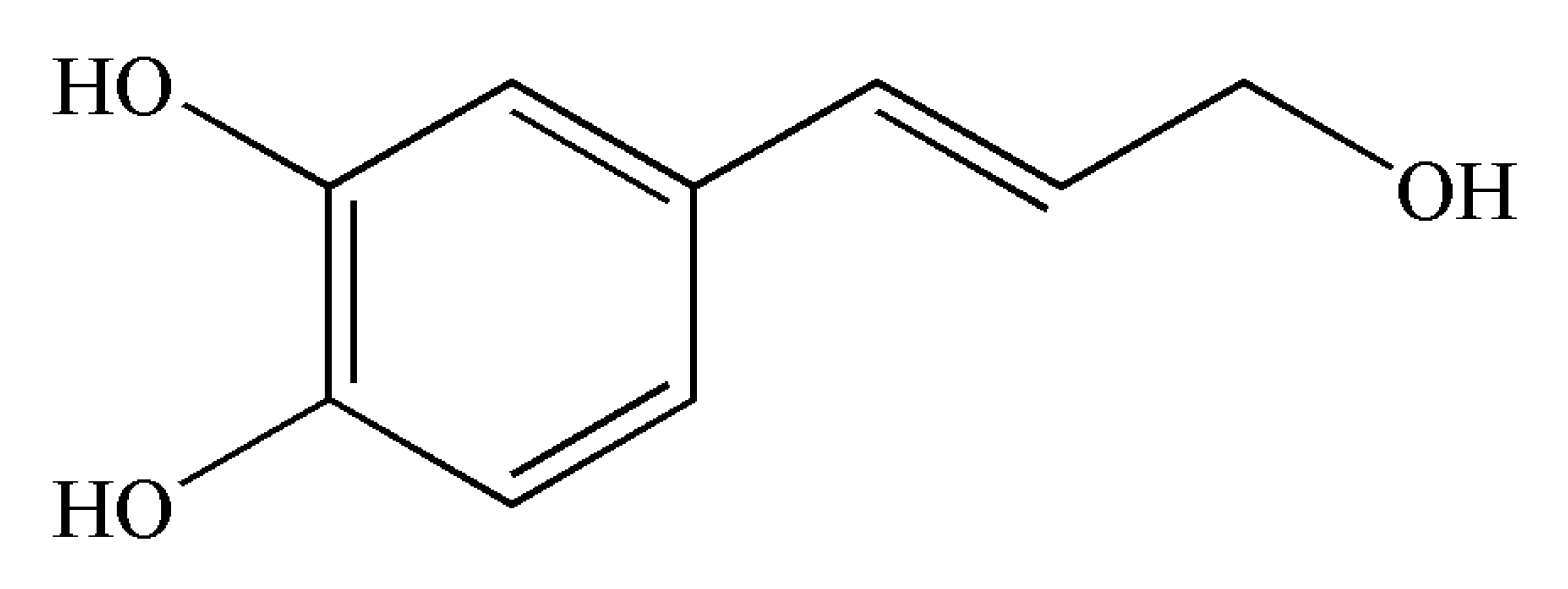
3.	Ferulic acid (FA, **3a**)	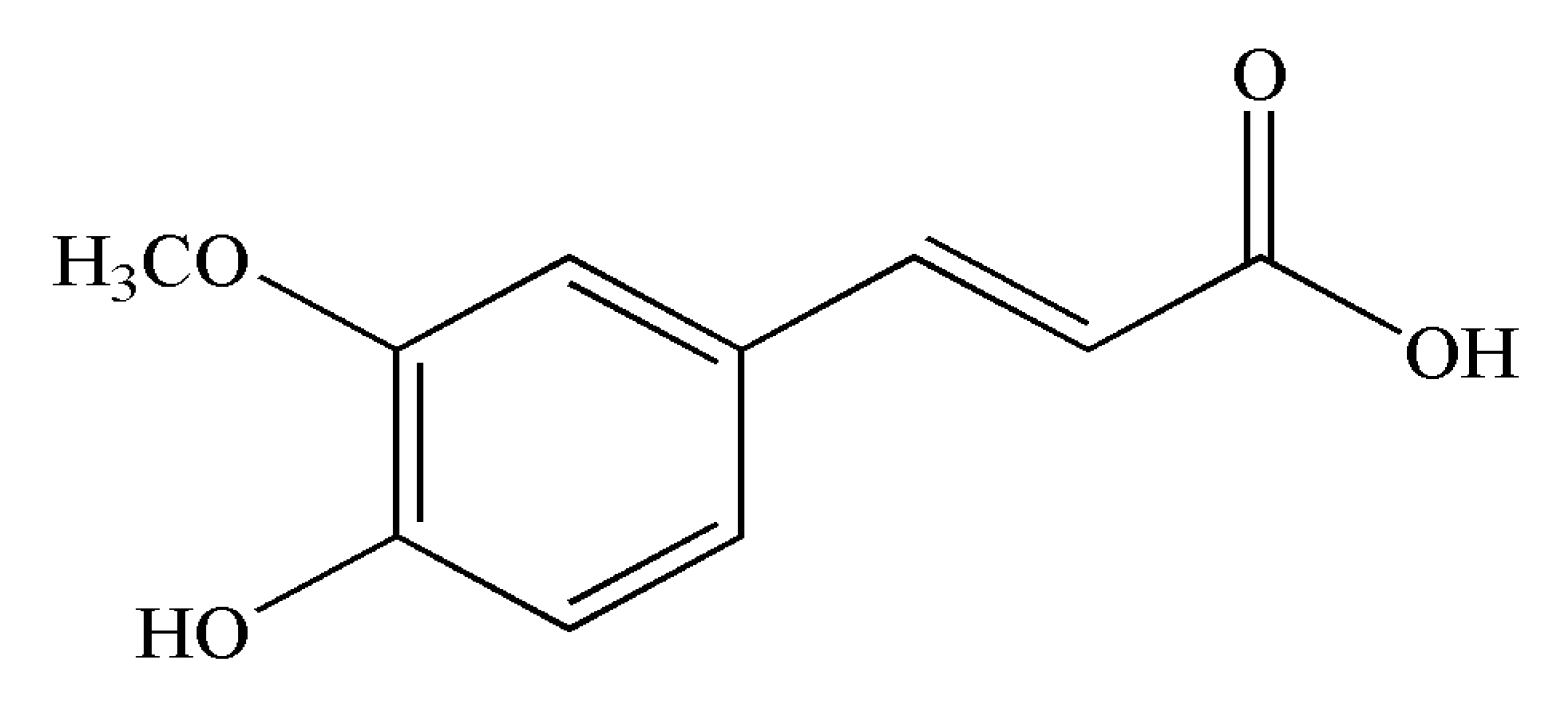	4-vinylguaiacol (4VG, **3b**)	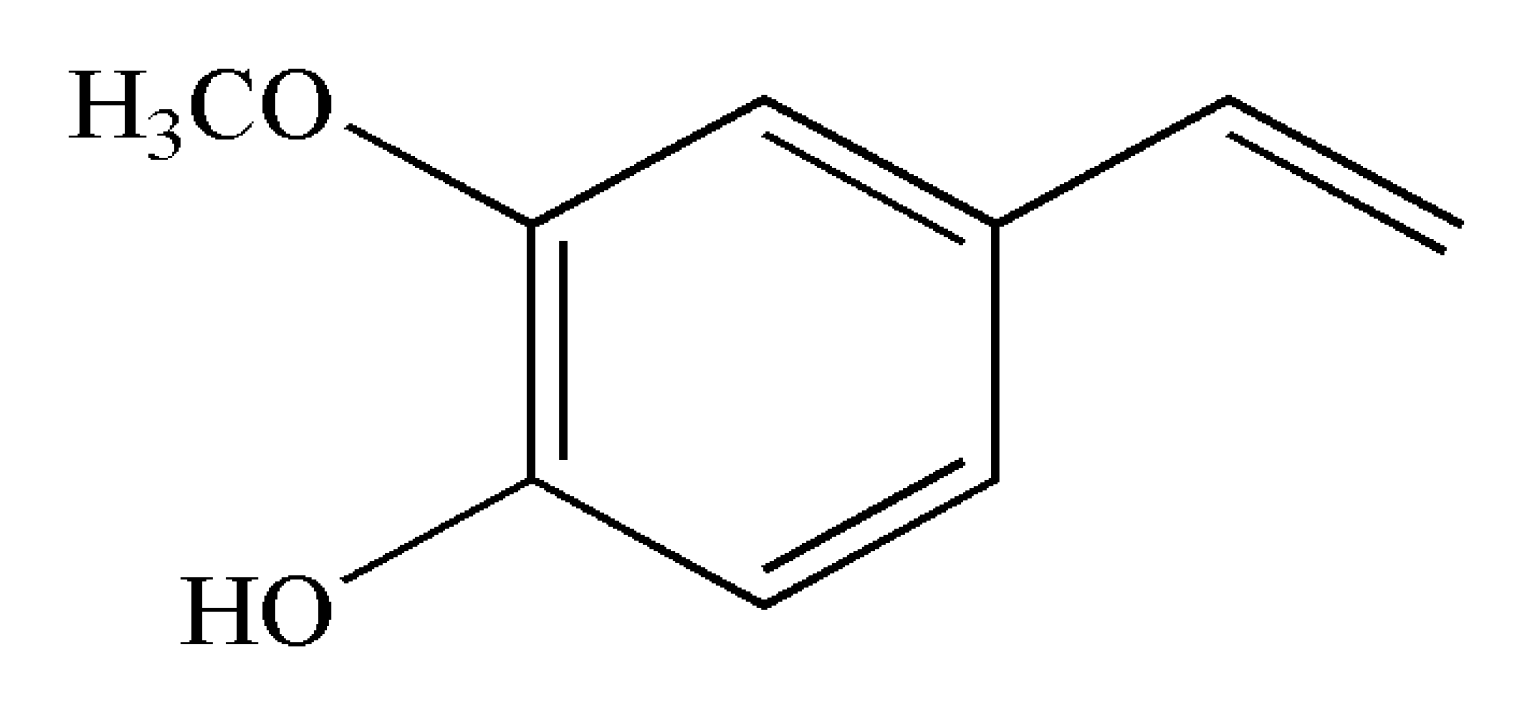	coniferyl aldehyde (**3c**)	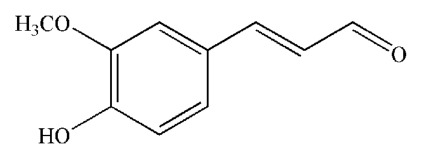	coniferyl alcohol (**3d**)	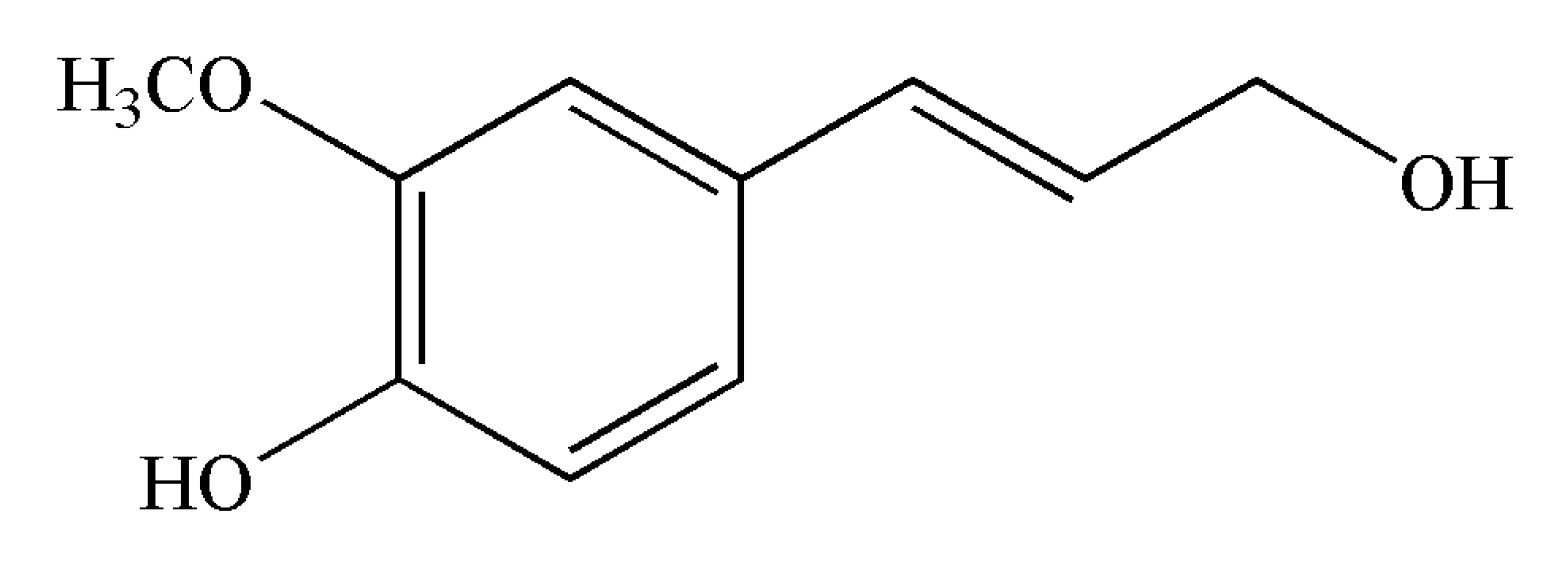
4.	Sinapic acid (SA, **4a**)	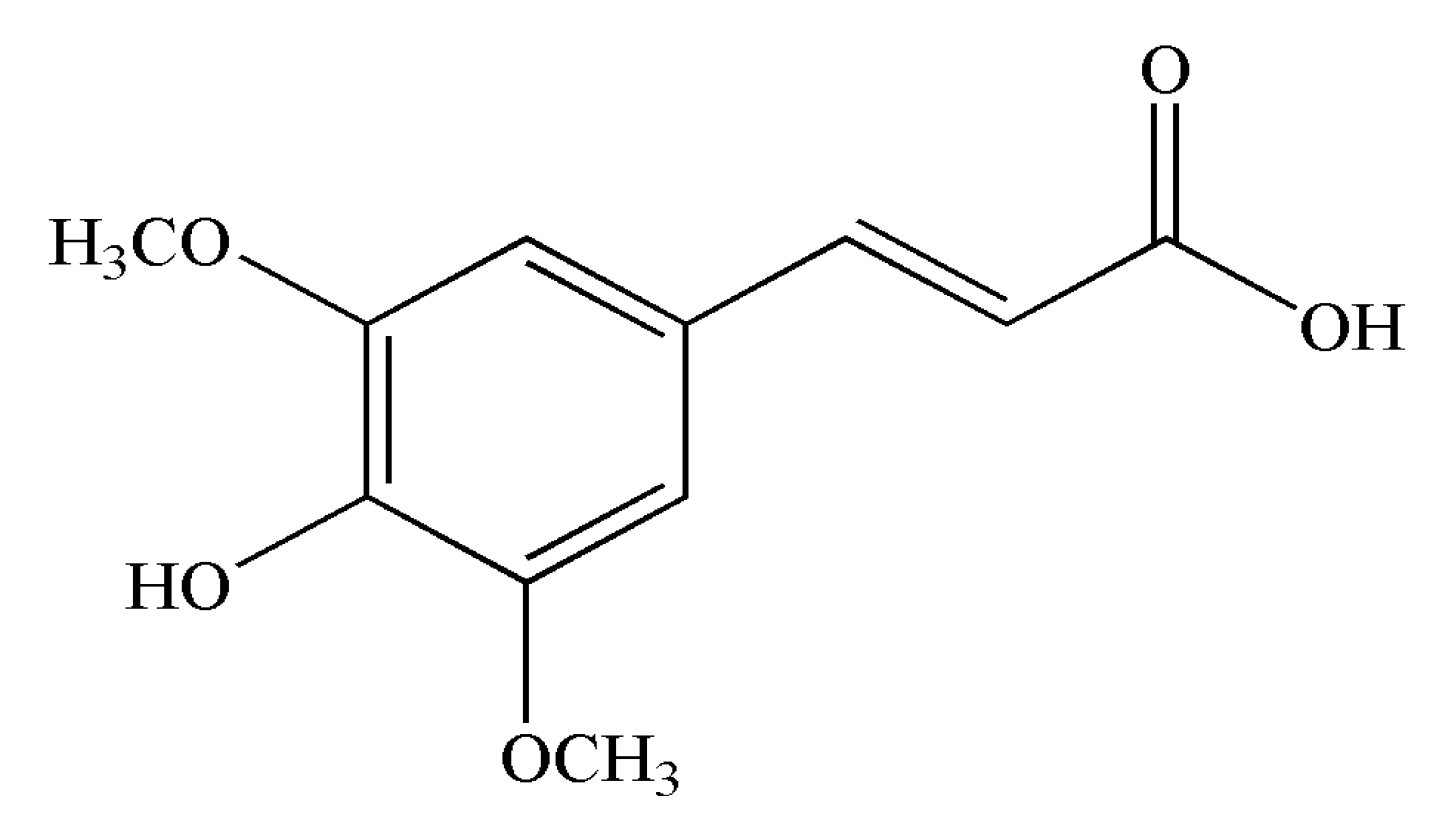	4-vinylsyringol (4VS, **4b**)	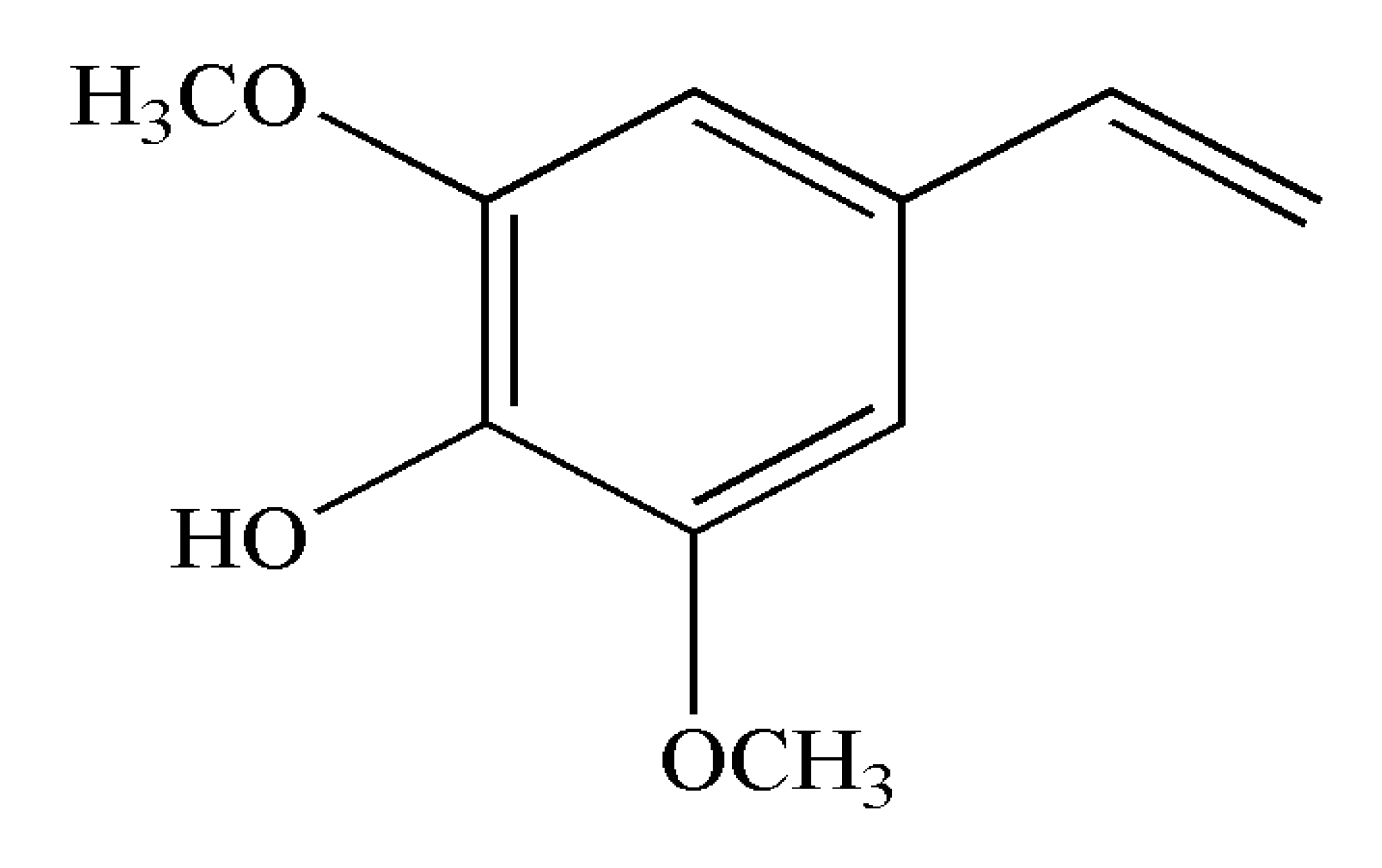	sinapyl aldehyde (**4c**)	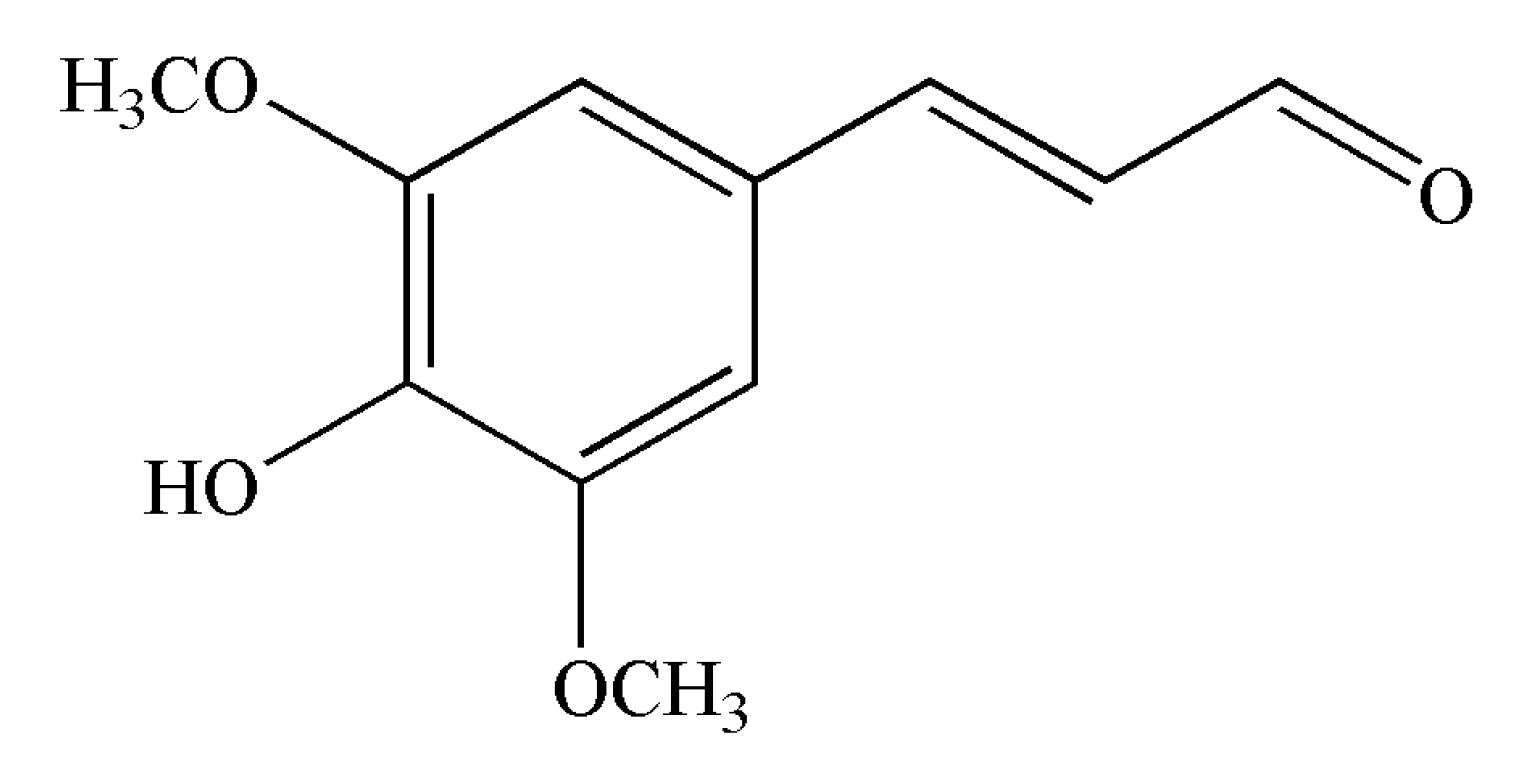	sinapyl alcohol (**4d**)	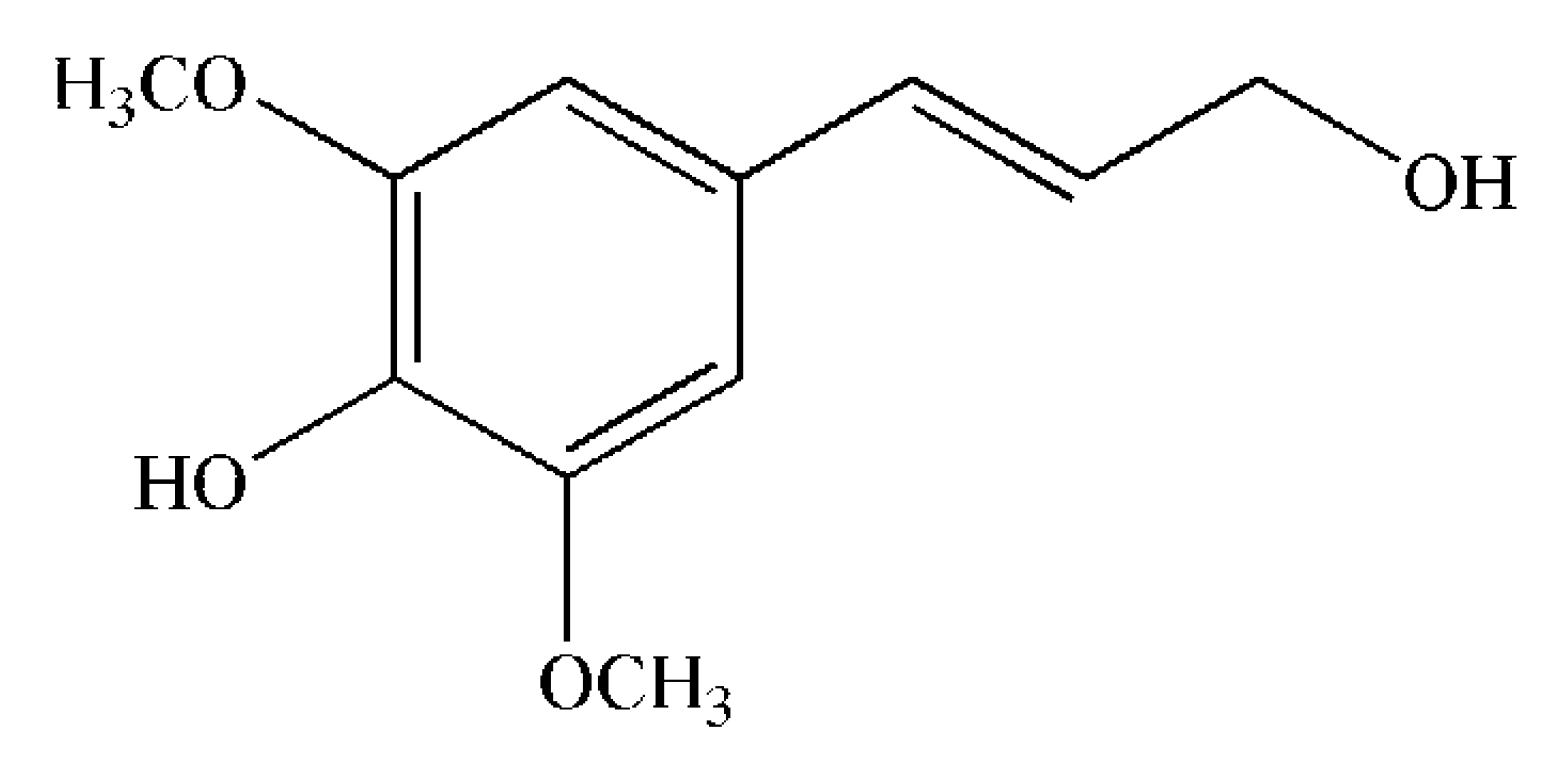

**Table 2:** Fungal strains selected for preparative-scale investigation based on their consistent PAD, CAR and/or ADH activity toward at least one substrate.

No.	Fungal Strain	Phenolic Acid Decarboxylase (PAD) Activity	Carboxylic Acid Reductase (CAR) Activity	Alcohol Dehydrogenase (ADH) Activity	Other Important Compounds
	**Endophytes**				
1.	*Aspergillus ochraceus* ROB-L1	No	No	No	Yes—vanillic or isovanillic acid
2.	*Aspergillus westerdijkiae* 17P	Yes (**2b** and **3b**)	Yes (**3c**)	Yes (**3d**)	No
3.	*Chaetomium cochliodes* KLON-L1	No	No	No	Yes—4-vinyl 1-cyclohexene diepoxide
4.	*Coniochaeta velutina* SW-B	Yes (**1b**)	No	No	No
5.	*Diaporthe eres* POP-L1	Yes (**1b** and **3b**)	Yes (**2c, 3c** and **4c**)	Yes (**1d, 2d, 3d** and **4d**)	No
6.	*Diaporte* sp. MOD-B	Yes (**1b** and **3b**)	Yes (**2c** and **4c**)	Yes (**1d, 2d** and **4d**)	No
7.	*Penicillium chrysogenum* SOS-B2	Yes (**1b** and **3b**)	No	No	No
8.	*Penicillium yarmokense* JAR-L1	Yes (**4b)**	No	No	Yes—vanillic or isovanillic acid, syringic acid, acetosyringone
9.	*Trichoderma harizanum* BUK-T	Yes (**1b, 2b, 3b** and **4b**)	Yes (**1c, 2c, 3c** and **4c**)	Yes (**1d, 2d, 3d** and **4d**)	Yes—syringic acid, acetosyringone
10.	*Umbelopsis* sp. 42	No	No	Yes (**3d**)	No
11.	*Umbelopsis* sp. JAR-T	Yes (**1b, 2b** and **3b**)	Yes (**4c**)	Yes (**4d**)	No
	**Basidiomycetes**				
12.	*Laetiporus sulphureus* AM 515	No	Yes (**1c, 2c, 3c** and **4c**)	Yes (**1d, 2d, 3d** and **4d**)	No
13.	*Phanerochaete chrysosporium* KKP 784	No	Yes (**1c, 2c, 3c** and **4c**)	Yes (**1d, 2d, 3d** and **4d**)	No
14.	*Trametes hirsuta* d28	No	Yes (**1c, 2c** and **3c**)	Yes (**1d, 2d** and **3d**)	No

**Table 3:** Biotransformation of *para*-coumaric acid (*p*CA, **1a**) by selected fungal strains. Values are expressed as percentages, where **1a** represents the substrate and **1b–1d** represent the corresponding biotransformation products. The percentage for **1a** indicates the remaining substrate after biotransformation, whereas the percentages for **1b–1d** indicate the yields of the respective biotransformation products.

Strain	Time (Days)	1a	1b	1c	1d	Other Metabolites
*Diaporthe* sp. MOD-B	1	19	15	-	61	5
	2	-	26	-	65	9
	3	-	36	-	58	6
	7	-	33	-	51	16
*P. chrysogenum* SOS-B2	2	41	47	-	12	-
	3	14	86	-	-	-
	6	15	78	-	7	-
	7	13	79	-	8	-
*Umbelopsis* sp. JAR-T	1	60	40	-	-	-
	2	10	88	-	2	-
	3	2	96	-	2	-
	7	-	98	-	2	-

**Table 4:** Biotransformation of caffeic acid (CA, **2a**) by selected fungal strains. Values are expressed as percentages, where **2a** represents the substrate and **2b–2d** represent the corresponding biotransformation products. The percentage for **2a** indicates the remaining substrate after biotransformation, whereas the percentages for **2b–2d** indicate the yields of the respective biotransformation products.

Strain	Time (Days)	2a	2b	2c	2d	Other Metabolites
*C. cochliodes* KLON-L1	2	49	31	-	11	9
	3	45	30	-	9	16
	6	35	26	-	7	32
	7	35	26	-	7	31
*Diaporthe* sp. MOD-B	1	69	-	-	4	27
	2	-	-	-	59	41
	3	-	-	-	71	29
	7	-	-	-	37	63

**Table 5:** Biotransformation of ferulic acid (FA, **3a**) by selected fungal strains. Values are expressed as percentages, where **3a** represents the substrate and **3b–3d** represent the corresponding biotransformation products. The percentage for **3a** indicates the remaining substrate after biotransformation, whereas the percentages for **3b–3d** indicate the yields of the respective biotransformation products.

Strain	Time (Days)	3a	3b	3c	3d	Other Metabolites
*A. westerdijkiae* 17P	1	88	8	-	4	-
	2	85	9	-	6	-
	5	-	11	8	75	6
*Diaporthe* sp. MOD-B	1	15	22	-	12	51
	2	-	14	-	10	76
	3	-	4	-	-	96
*P. chrysogenum* SOS-B2	1	63	15	-	22	-
	2	50	32	-	18	-
	5	26	28	-	46	-
*Umbelopsis* sp. JAR-T	1	6	94	-	-	-
	2	-	100	-	-	-
	3	-	100	-	-	-
	7	-	100	-	-	-

**Table 6:** Biotransformation of sinapic acid (SA, **4a**) by selected fungal strains. Values are expressed as percentages, where **4a** represents the substrate and **4b–4d** represent the corresponding biotransformation products. The percentage for **4a** indicates the remaining substrate after biotransformation, whereas the percentages for **4b–4d** indicate the yields of the respective biotransformation products.

Strain	Time (Days)	4a	4b	4c	4d	Other Metabolites
*T. harzianum* BUK-T	2	-	-	-	47	53
	3	-	-	-	52	48
	6	-	-	-	69	31
	7	-	-	-	68	32

## Data Availability

The datasets used and/or analyzed during the current study are available from the corresponding authors on reasonable request.

## Supplementary Materials

The following supporting information can be downloaded at: https://www.mdpi.com/article/10.3390/molecules31152609/s1, Figure S1: UHPLC-DAD chromatograms of reference standards relevant to *p*CA biotransformation. Figure S2: UHPLC-DAD chromatograms of reference standards relevant to CA biotransformation. Figure S3: UHPLC-DAD chromatograms of reference standards relevant to FA biotransformation. Figure S4: UHPLC-DAD chromatograms of reference standards relevant to SA biotransformation. Figure S5: (a–c): UHPLC-DAD chromatograms of *p*CA biotransformation by *Umbelopsis* sp. JAR-T in triplicates. Figure S6: (a–c): UHPLC-DAD chromatograms of FA biotransformation by *Umbelopsis* sp. JAR-T in triplicates. Figure S7: UHPLC-DAD chromatogram of final product 4-vinylphenol biotransformed from para-coumaric acid by *Umbelopsis* sp. JAR-T. Figure S8: UHPLC-DAD chromatogram of final product 4-vinylguaiacol biotransformed from ferulic acid by *Umbelopsis* sp. JAR-T.
