# Supplementary material for: Whole-Cell Fungal Biotransformation of para-Hydroxycinnamic Acids Mediated by Phenolic Acid Decarboxylase, Carboxylic Acid Reductase and Alcohol Dehydrogenase
Source: Molecules. 2026 Jul 27;31(15):2609. doi: 10.3390/molecules31152609 (PMC13468669; doi:10.3390/molecules31152609)

Supplementary materials

Whole-Cell Fungal Biotransformation of para-Hydroxycinnamic Acids mediated by Phenolic Acid Decarboxylase, Carboxylic Acid Reductase and Alcohol Dehydrogenase

Abirami Baskaran[1,*], Stefano Serra[2], El-Sayed R. El-Sayed[1,3], Tomasz Tronina[1], Jacek Łyczko[1], Teresa Olejniczak[1], Elisabetta Brenna[4], Filip Boratyński[1,*]

Figure S1. UHPLC-DAD chromatograms of reference standards relevant to *p*CA biotransformation.

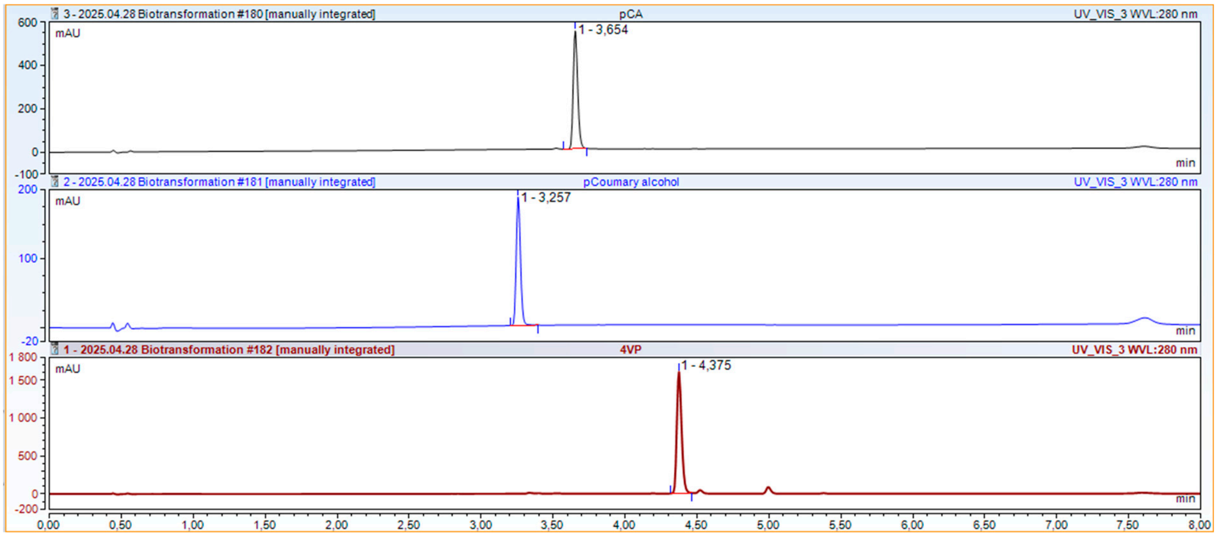

Figure S2. UHPLC-DAD chromatograms of reference standards relevant to CA biotransformation.

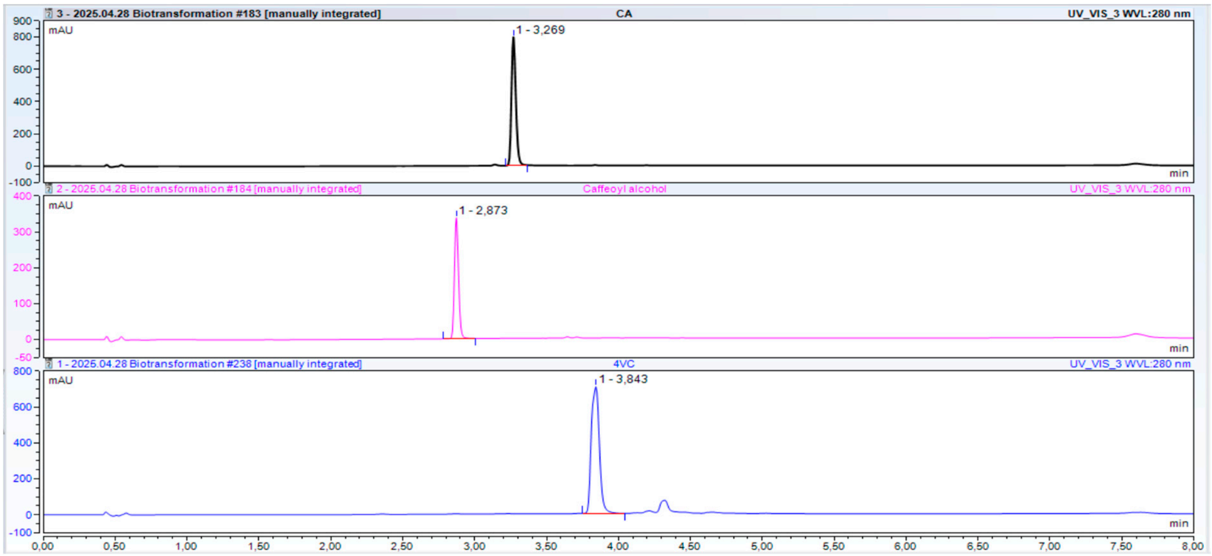

Figure S3. UHPLC-DAD chromatograms of reference standards relevant to FA biotransformation.

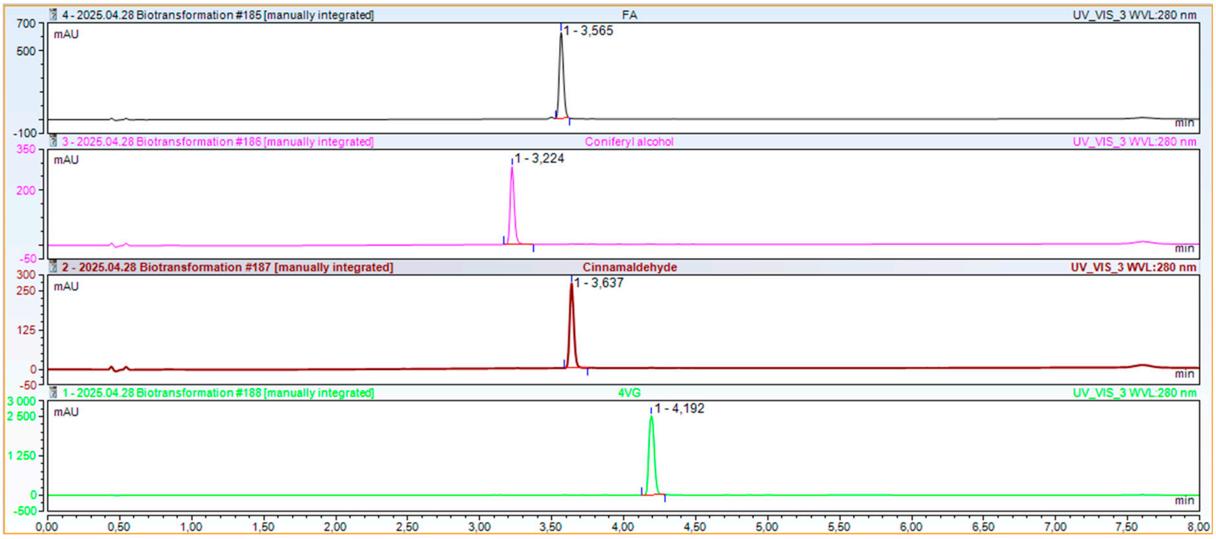

Figure S4. UHPLC-DAD chromatograms of reference standards relevant to SA biotransformation.

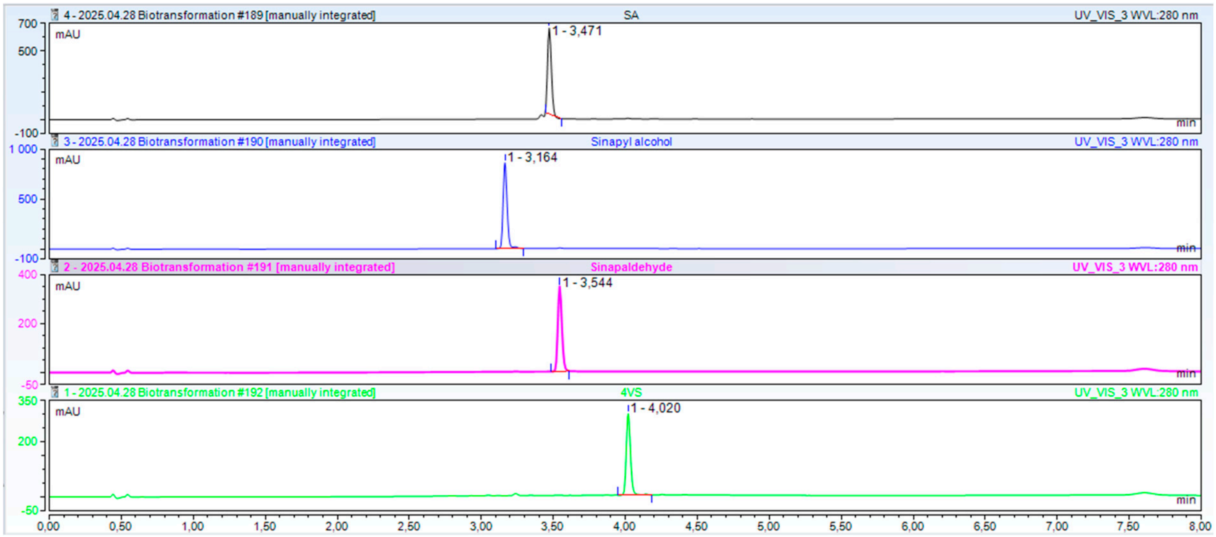

Figure S5. (a–c): UHPLC-DAD chromatograms of *p*CA biotransformation by *Umbelopsis* sp. JAR-T in triplicates.

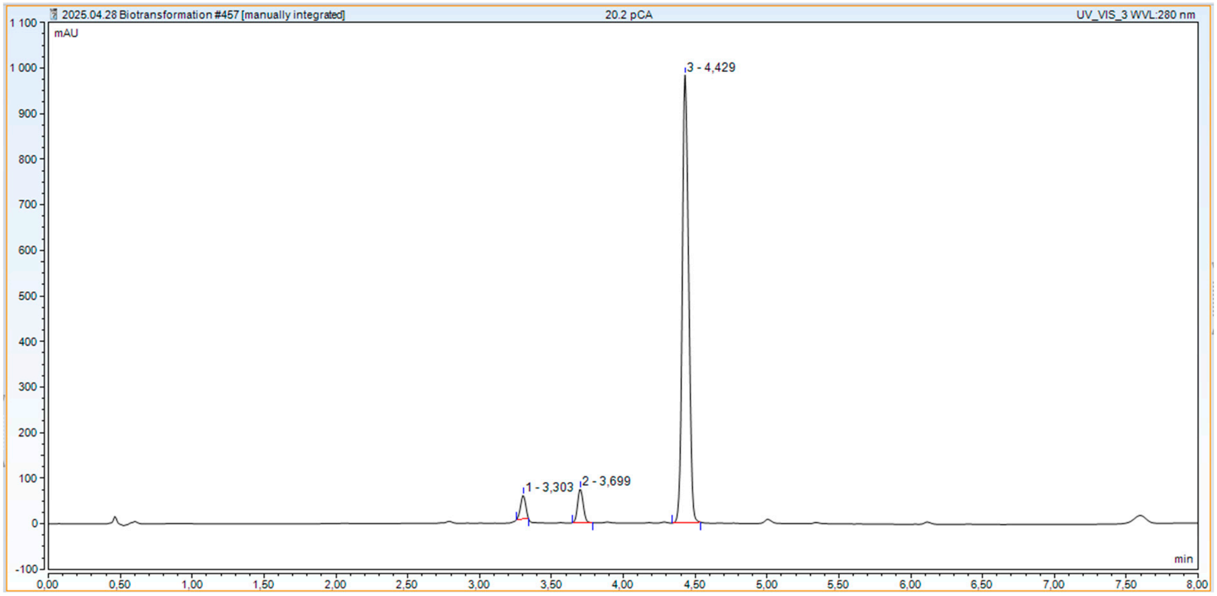

a.

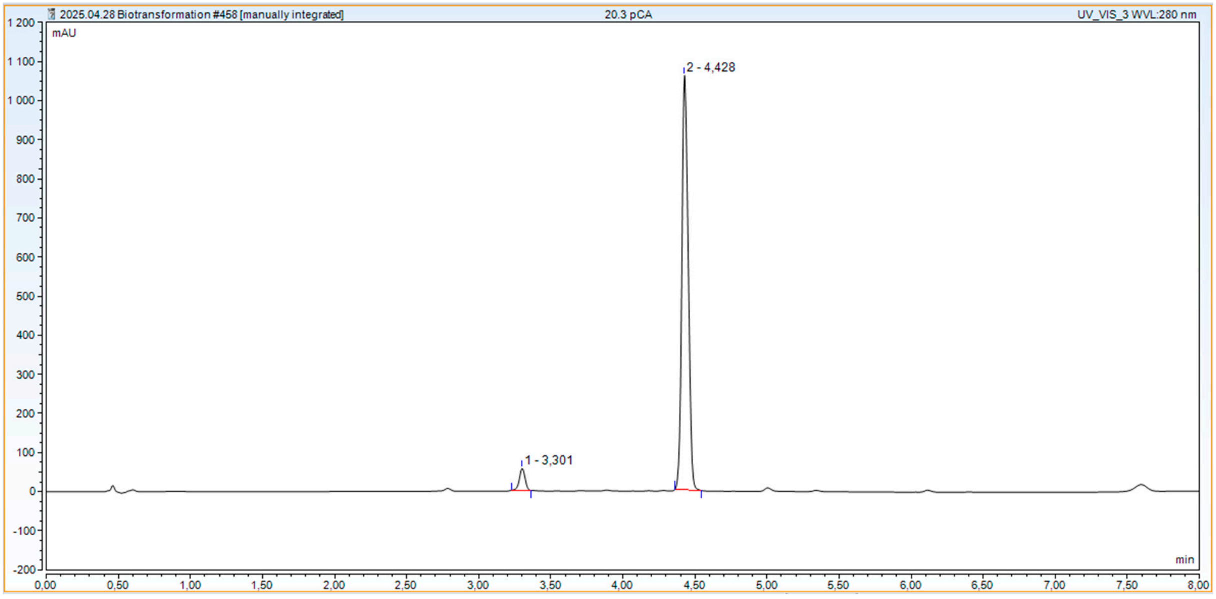

b.

c. 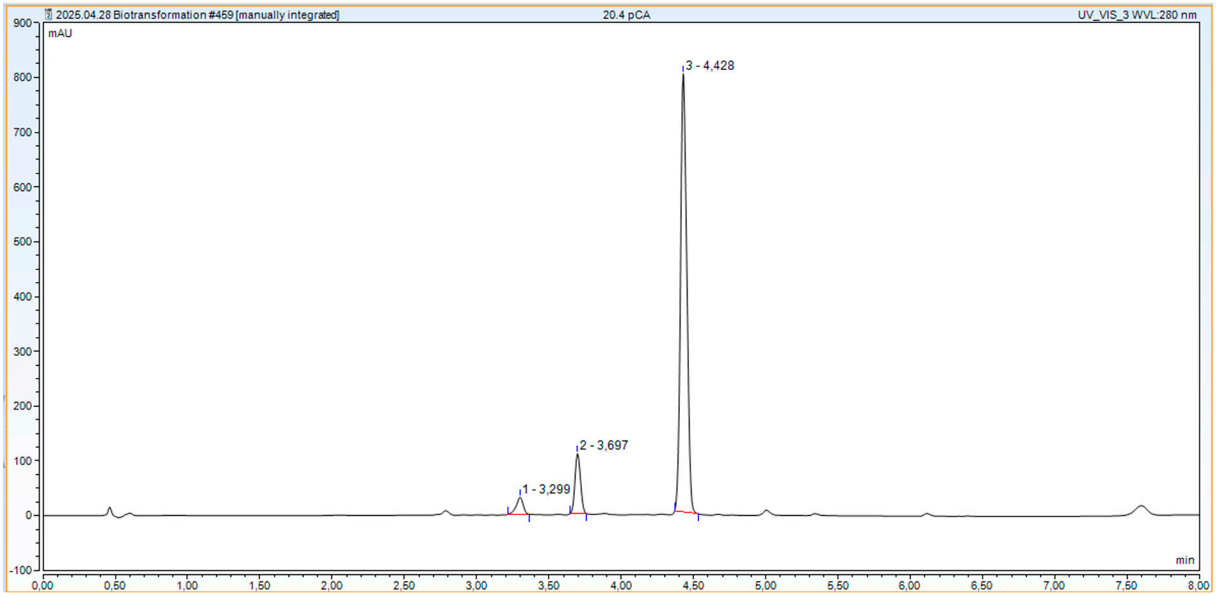
2025.04.28 Biotransformation #459 [manually integrated]
20.4 pCA
UV_VIS_3 WVL:280 nm
mAU
min
1 - 3,299
2 - 3,697
3 - 4,428

Figure S6. (a–c): UHPLC-DAD chromatograms of FA biotransformation by *Umbelopsis* sp. JAR-T in triplicates.

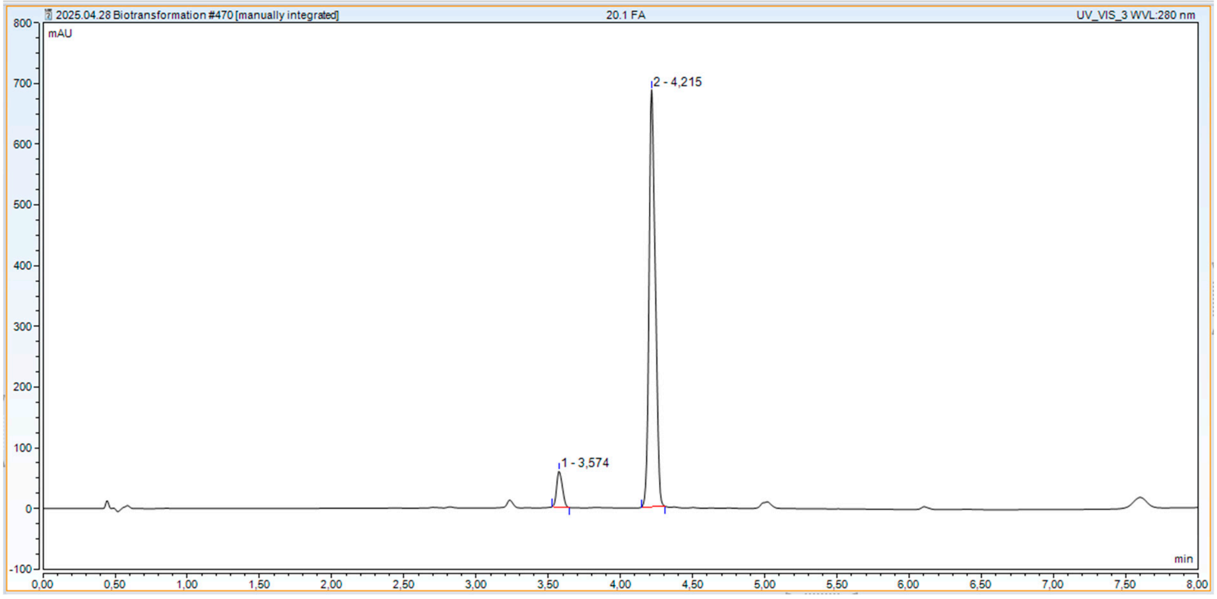

a.

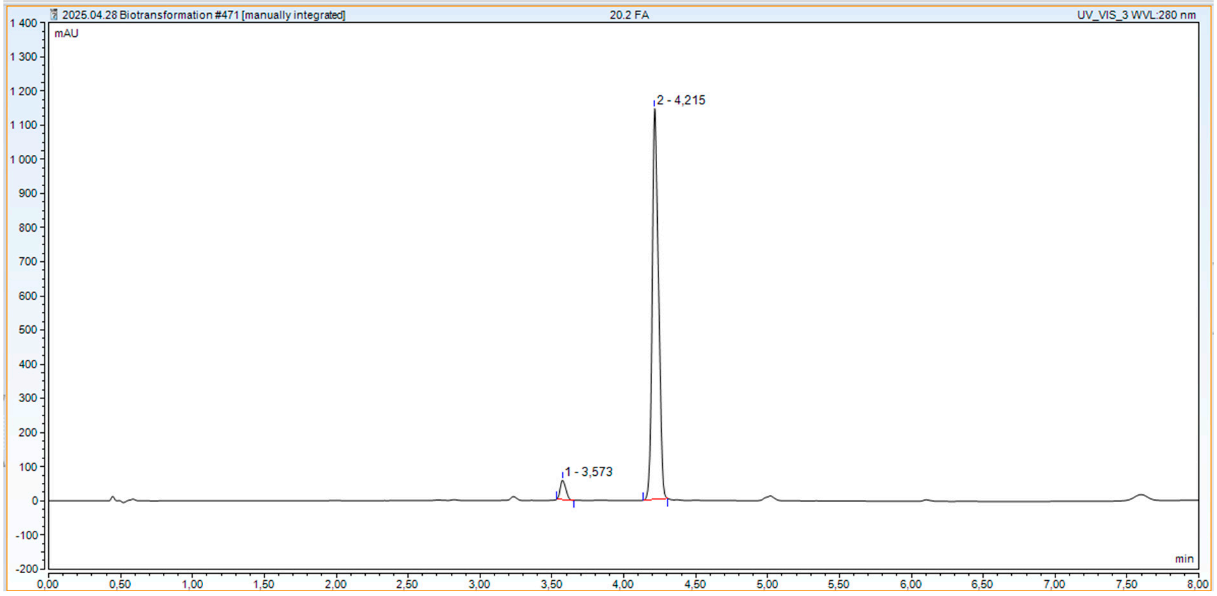

b.

c. 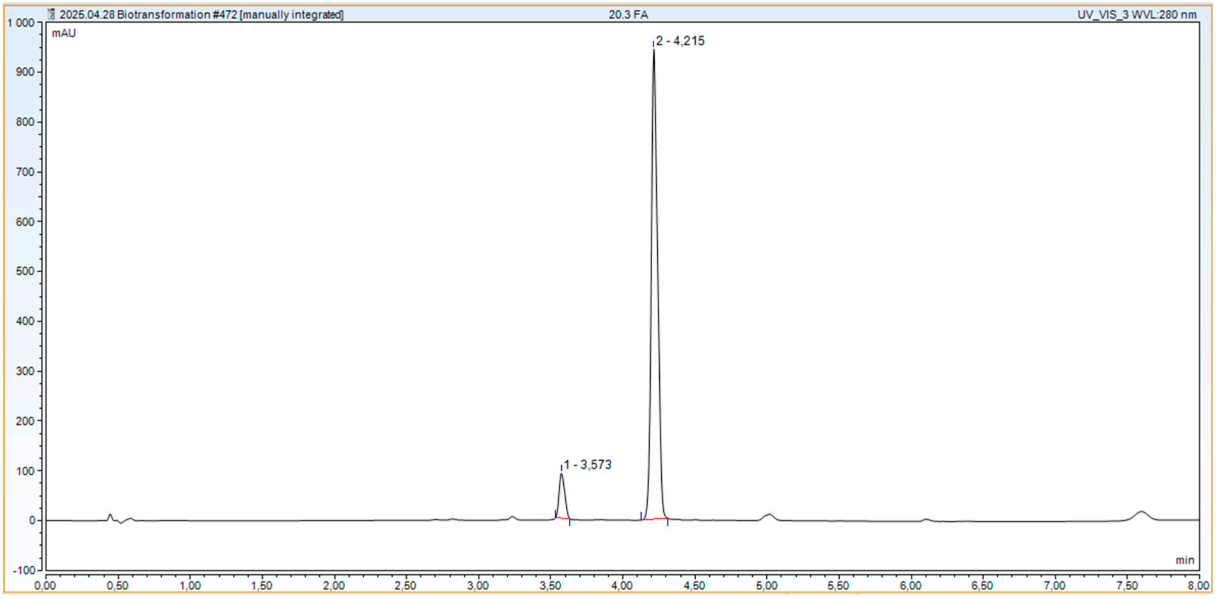
2025.04.28 Biotransformation #472 [manually integrated]
20.3 FA
UV_VIS_3 WVL:280 nm
mAU
1 - 3,573
2 - 4,215
min

Figure S7. UHPLC-DAD chromatogram of final product 4-vinylphenol biotransformed from *para*-coumaric acid by *Umbelopsis* sp. JAR-T.

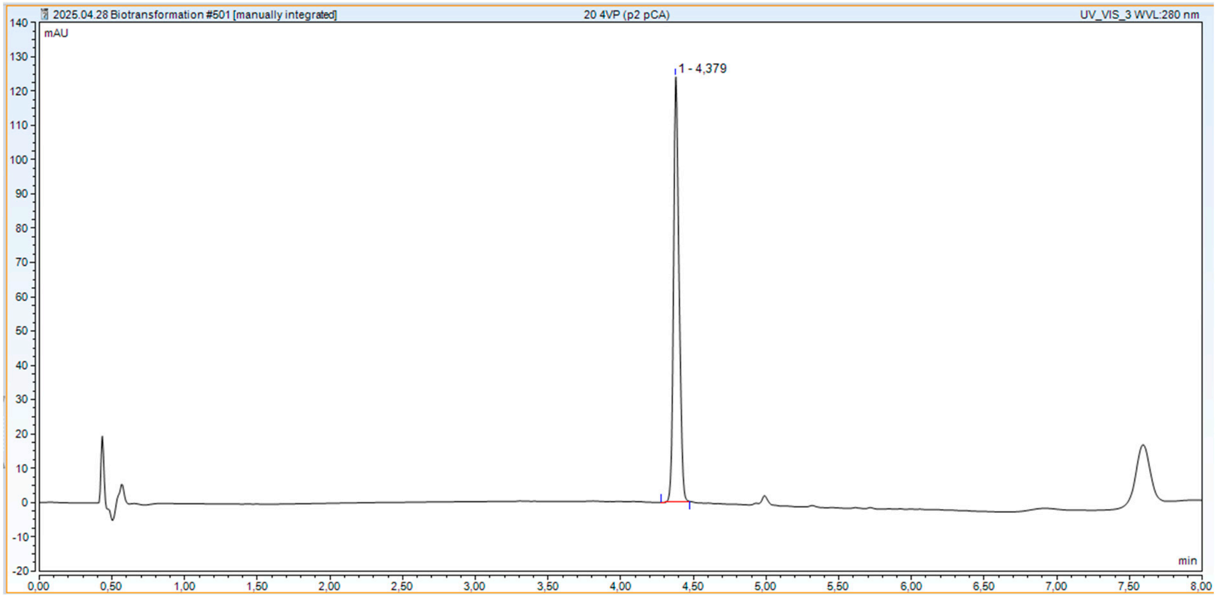

Figure S8. UHPLC-DAD chromatogram of final product 4-vinylguaiacol biotransformed from ferulic acid by *Umbelopsis* sp. JAR-T.

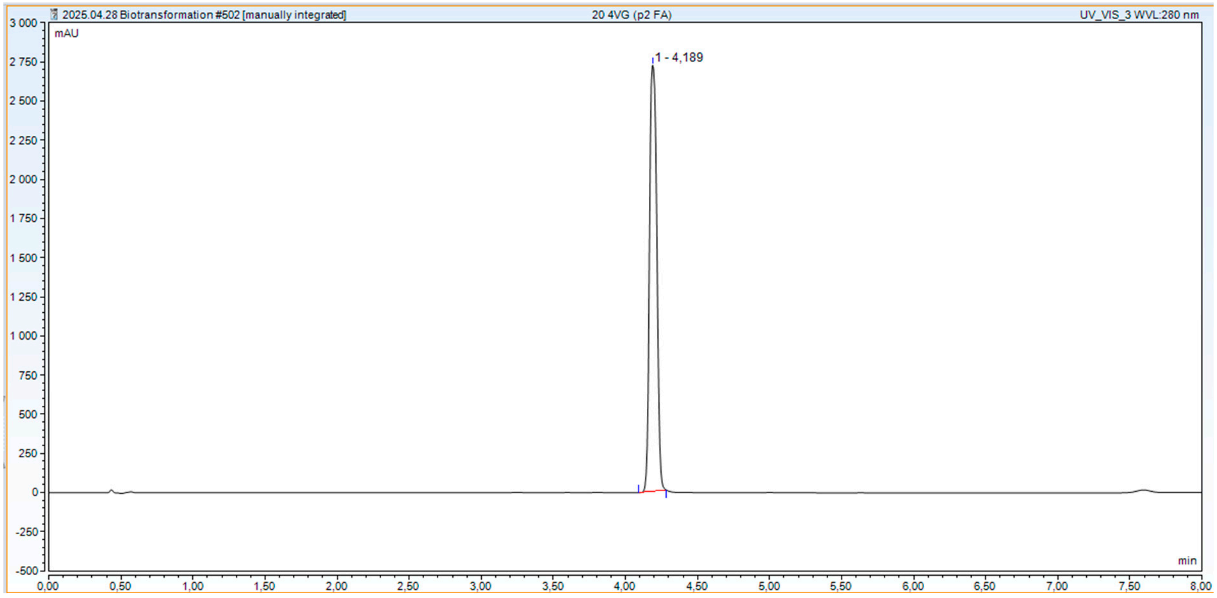

Supplement: Supplementary file 1 [file molecules-31-02609-s001.zip › molecules-4416493-supplementary.pdf]
